# Simultaneous Detection of Neural Activity and Temperature in Photothermal Neural Stimulation

**DOI:** 10.1002/advs.202411725

**Published:** 2025-03-26

**Authors:** Duhee Kim, Jee Woong Lee, Seoyoung Kang, Woongki Hong, Jungha Lee, Hyuk‐Jun Kwon, Jae Eun Jang, Luke P. Lee, Hongki Kang

**Affiliations:** ^1^ Department of Electrical Engineering and Computer Science Daegu Gyeongbuk Institute of Science and Technology (DGIST) Daegu 42988 Republic of Korea; ^2^ Department of Bioengineering Department of Electrical Engineering and Computer Sciences University of California at Berkeley Berkeley CA 94720 USA; ^3^ School of Undergraduate Studies College of Transdisciplinary Studies Daegu Gyeongbuk Institute of Science and Technology (DGIST) Daegu 42988 Republic of Korea; ^4^ Renal Division and Division of Engineering in Medicine Department of Medicine Brigham and Women's Hospital Harvard Medical School Boston MA 02115 USA; ^5^ Institute of Quantum Biophysics Department of Biophysics Sungkyunkwan University Suwon 16419 South Korea; ^6^ Department of Biomedical Engineering Seoul National University College of Medicine Seoul 03080 Republic of Korea; ^7^ Interdisciplinary Program in Bioengineering College of Engineering Seoul National University Seoul 08826 Republic of Korea; ^8^ Seoul National University Hospital Seoul 03080 Republic of Korea; ^9^ Institute of Medical and Biological Engineering Medical Research Center Seoul National University Seoul 03080 Republic of Korea

**Keywords:** high‐resolution direct temperature sensing, low‐noise neural electrodes, multifunctional microelectrode array, Photothermal neuromodulation, Transparent ultrathin Au

## Abstract

Photothermal neuromodulation is a promising non‐electrical neural stimulation technology for treating brain diseases through optically induced cell membrane temperature changes. However, the technology faces limitations in understanding its mechanism and impact on cellular behavior due to the restriction of directly measuring temperature changes at the cell interface from a very close distance during optical stimulation of neural cells, necessitating advancements in high‐precision temperature sensing and electrical recording without light interference. This challenge is addressed by developing ultrasensitive cell membrane interface temperature sensors integrated with low‐noise electrical recording capabilities. Transparent resistive temperature detectors, composed of a 10 nm thickness of ultrathin Au film fabricated by polyelectrolyte seed layer‐induced thermal evaporation, achieved precise measurement and control of temperature changes without significant light interference and self‐heating. A transparent electrode composed of the same ultrathin Au layer shows low‐noise electrical recordings of neural signals upon photothermal stimulation. Using this multifunctional system, it is demonstrated that an average increase of 2.34 °C at neuronal cell surfaces results in over 95% suppression of hippocampal neural spike activities. The approach provides unprecedented insights into the mechanisms of photothermal neuromodulation and its effects on cellular behavior, paving the way for advanced treatments of neurological disorders.

## Graphical abstract

Photothermal neuromodulation technology faces limitations in understanding its mechanisms and effects on cellular behavior due to the difficulty of directly measuring temperature changes at the cell interface during optical stimulation of neurons. To overcome this challenge, ultrasensitive, transparent temperature sensors integrated with low‐noise electrical recording capabilities are presented. As a result, temperature change‐dependent photothermal neural inhibition is successfully demonstrated.

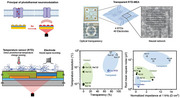

## 1 Introduction

Photothermal neuromodulation techniques have shown great promise in optically modulating brain activities without genetic modification for neurological disorder treatments such as epilepsy or seizure.^[^ ^1^, ^2^, ^3^, ^4^, ^5^, ^6^, ^7^, ^8^, ^9^, ^10^ ^]^ Photothermal neural stimulation induces highly localized and transient temperature changes in cell membranes coupled with light‐stimulation reactive nanomaterials.^[^ ^11^ ^]^ The temperature changes can affect the electrical characteristics of the cell membrane or temperature‐sensitive ion channels.^[^ ^12^, ^13^ ^]^ Temperature is a stimulus that can cause biological changes, and organisms respond to temperature changes in various ways to maintain homeostasis.^[^ ^14^, ^15^ ^]^ Temperature‐sensitive ion channels play an important role in cellular physiology including neurons, as molecular thermometers that translate thermal stimuli into dynamic changes in membrane potential.^[^ ^16^, ^17^, ^18^, ^19^, ^20^, ^21^, ^22^ ^]^ The closer to the cell surface temperature changes in response to photothermal stimulation are measured, the more accurately the mechanism for regulating cell activity will be understood. Therefore, the cell interface temperature change at which the temperature‐sensitive ion channel responds still needs to be discovered. Over the past decade, most of the research on photothermal neural modulation has reported the laser power density with the neural stimulation effect due to the lack of the ability to directly measure the cell interface temperature during the optical stimulation. Thus, it has not succeeded in carefully discovering the cell network interface temperature changes during neuromodulation.

For example, the IR camera‐based temperature sensing shows inaccuracy because the temperature is measured primarily on the surface of cell culture media, not on the cell interface.^[^ ^4^, ^8^, ^23^ ^]^ Additionally, real‐time cell interface temperature sensing with an IR camera is impossible because the temperature of the entire cell culture media rises slowly. Several electrical temperature sensors have been introduced to measure cell interface temperature directly, including thermistors,^[^ ^24^ ^]^ resonators,^[^ ^25^ ^]^ thermocouples,^[^ ^26^ ^]^ etc.^[^ ^27^ ^]^ However, these temperature sensors still suffer from self‐heating upon light illumination or poor linearity. Several studies have explored the measurement of temperature fluctuations induced by optical stimulation using penetration neural probes in vivo.^[^ ^28^, ^29^, ^30^ ^]^ However, the temperature measurements were only performed with distance due to the possibility of the self‐heating.

On the other hand, the resistive temperature detector (RTD) will be a great alternative because of its simplest structure and sensing mechanism.^[^ ^31^, ^32^, ^33^ ^]^ Thus, RTD can show rapid response, enabling real‐time high‐speed sensing. Recently, non‐transparent Pt or Ni‐based RTDs have been suggested to sense the temperature of the photothermal layer interface when performing photothermal heat‐induced photonic Polymerase Chain Reaction (PCR).^[^ ^32^ ^]^ However, there are still remaining problems that the opaque temperature sensors either reflect or absorb the light, resulting in significant light interference and inaccurate temperature measurement during the photothermal neuromodulation

Alternatively, indirect nanoparticle‐based optical temperature sensors have also been reported for detecting cell interface temperature using diamond nanoparticles,^[^ ^34^, ^35^ ^]^ fluorescence proteins,^[^ ^36^, ^37^ ^]^ quantum dots,^[^ ^38^, ^39^ ^]^ metallic nanoparticles, and nanorods.^[^ ^40^, ^41^ ^]^ These temperature sensors are more appropriate for single‐cell level temperature sensing than monitoring neural modulation on the network level in a larger area. In addition, these optical temperature sensing methods are more suitable for short‐term temperature monitoring environments because these particles show degradation due to their time‐dependent material characteristics, such as photobleaching.

In this work, we propose a transparent RTD‐Microelectrode array (tRTD‐MEA) to photothermally modulate neural cell signals while directly measuring the temperature changes and electrical signals of the cells through tRTD‐MEA. The electrodes and temperature sensors were composed of transparent 10 nm‐thick ultrathin Au film fabricated by polyelectrolyte seed layer‐induced thermal evaporation, which shows excellent conductivity, transparency, and biocompatibility. Additional layers of Au island structures and transparent conductive polymer (Poly(3,4‐ethylene dioxythiophene):poly(styrene sulfonate) (PEDOT:PSS) allow transparent electrodes to show excellent electrochemical properties while implementing the photothermal effect with near‐infrared (NIR) light. The transparent temperature sensors accurately monitored the temperature change of neural cells in the cell culture medium conditions. With this tRTD‐MEA, we successfully demonstrated optical imaging and neural recording, direct sensing of photothermal heat‐induced temperature changes, and photothermal neural inhibitory stimulation. We detected the temperature changes on the cell surface for photothermal neural inhibitory stimulation on the tRTD‐MEA to discover how much temperature change occurs to activate temperature‐sensitive ion channels. As a result, our temperature‐based neural signal regulation can provide a thermal standard for other studies of optical stimulation with different light sources and conditions. Moreover, measuring temperature changes directly at the microscale of neural cells will be an important indicator in developing treatment methods for neural diseases.

## 2 Results

### 2.1 Concepts and Device Structures of Transparent RTD‐MEA

**Figure 1:**
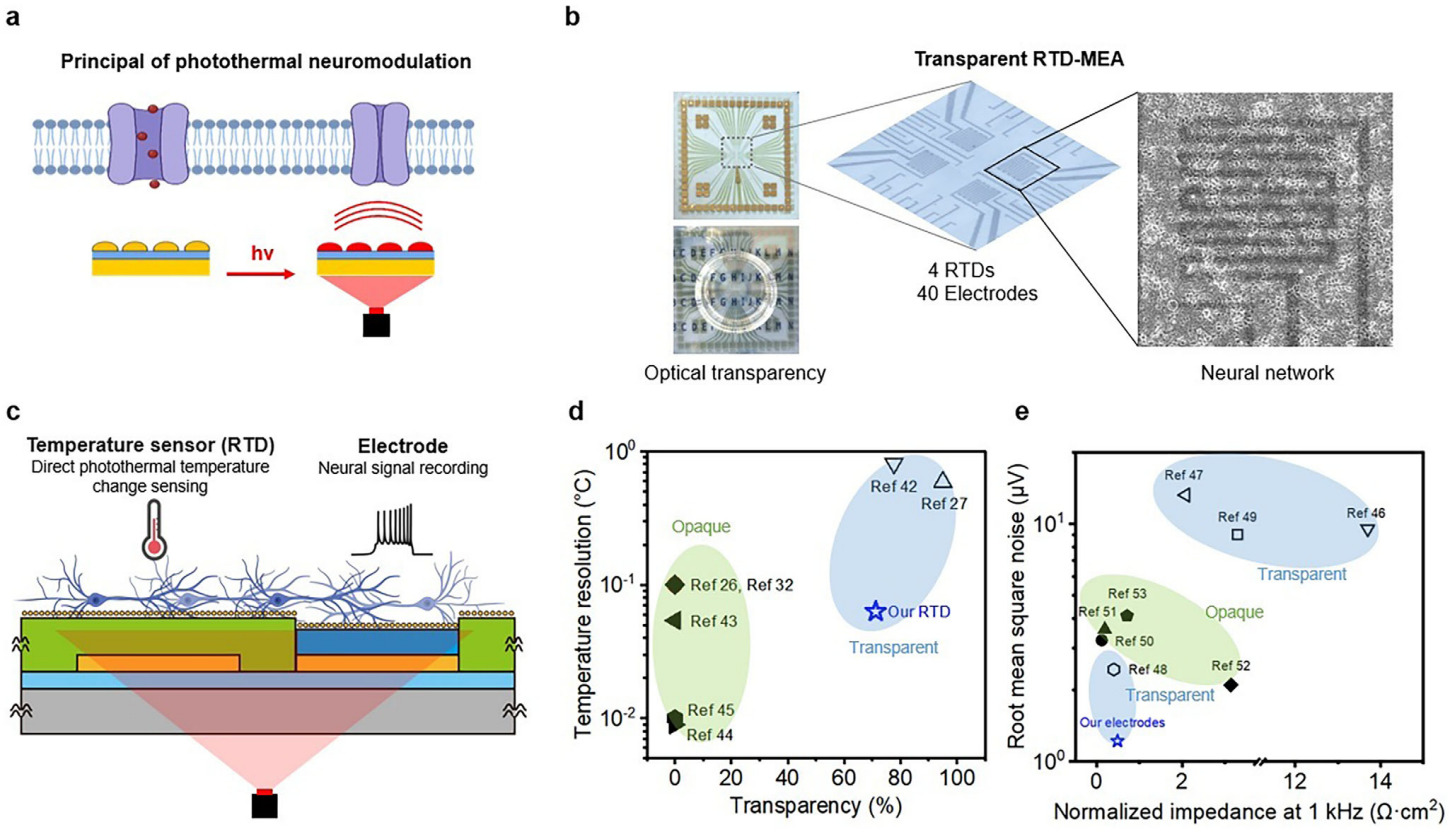
Concepts and device structures of transparent RTD‐MEA (tRTD‐MEA). a) Schematic of controlling thermosensitive ion channels with bottom‐located photothermal‐induced heat. b) Optical images of tRTD‐MEA, including 4 sets of temperature sensors (RTDs) with cultured neural networks and 40 channels of microelectrodes. c) Proposed operation of tRTD‐MEA: Simultaneous direct sensing of photothermal temperature changes and electrophysiological signal recording during photothermal neural stimulation. d) Comparison of temperature sensing resolution and optical transparency with other transparent/opaque direct temperature sensors proposed in bioengineering applications. Transparent temperature sensors are marked as hollow symbols grouped in sky blue color,^[^ ^27^, ^42^ ^]^ (our temperature sensor) and opaque temperature sensors are marked as solid symbols grouped in light green color.^[^ ^26^, ^32^, ^43^, ^44^, ^45^ ^]^ **e)** Comparison of area‐normalized impedance and RMS noise with other electrodes. Transparent electrodes are marked as hollow symbols grouped in sky blue color,^[^ ^46^, ^47^, ^48^, ^49^ ^] (^our electrode) and opaque electrodes are marked as solid symbols grouped in light green color.^[^ ^50^, ^51^, ^52^, ^53^ ^]^

Previous studies on photothermal neural stimulation have reported both excitation and inhibition of neural activities depending on the intensity of the stimulating light, but have yet to report cell interface temperature changes due to limitations in temperature sensing methods.^[^ ^4^, ^6^, ^8^, ^9^, ^10^, ^23^ ^]^ In contrast to previous research, we utilized tRTD‐MEA to investigate the relationship between inhibition rate and cell interface temperature changes. Irradiation with a 785‐nm wavelength NIR laser induces rapid localized heating in the Au island (4 nm) photothermal layer of the tRTD‐MEA, potentially leading to the closure of thermosensitive ion channels and inhibition of neural activity (**Figure** **1**a).

The tRTD‐MEA consists of 4 resistance temperature detectors (RTD) and a 40‐channel MEA, with Au islands serving as the photothermal layer (Figure 1b; Figure S1a,b, Supporting Information). First, bottom‐inserted transparent ultrathin Au (10 nm)/polyelectrolyte multilayer (PEM) temperature sensors monitor temperature changes induced by photothermal heat transfer to the cells. Second, transparent, low‐impedance electrodes spanning 40 channels consist of Au islands (4 nm)/PEDOT:PSS (15 nm)/ultrathin Au (10 nm)/PEM to record neural spike signals (Figure S1c–f, Supporting Information). Third, a top‐located photothermal layer comprises independent 4 nm‐thick Au islands above the parylene‐C passivation layer. This photothermal layer can induce rapid and localized temperature changes during near‐infrared (NIR) laser irradiation.^[^ ^8^, ^42^ ^]^ We selected a 4 nm thickness for the photothermal layer as optimal, based on our previous work indicating maximum light‐to‐heat conversion efficiency at deposition thicknesses of 3–5 nm.^[^ ^8^ ^]^ However, after Au deposition, the Au particles partially connected as a thin film rather than remaining completely independent as Au islands, due to strong adhesion between Au and parylene‐C, resulting in partially resistive properties (Figure S2, Supporting Information). To address this, we implemented a simple heat treatment (150 °C for 20 min) across the entire device on a hot plate, causing the partially connected Au thin film to completely dewetted into discrete Au islands (Figure S2, Supporting Information). The overall fabrication process of the tRTD‐MEA is detailed in Figure S3 (Supporting Information). With the tRTD‐MEA, we can quantify and correlate neural stimulation rates with cellular interface temperature changes during thermal neuromodulation (Figure 1c). Furthermore, leveraging the transparency of the tRTD‐MEA enables optical imaging and calcium recording while eliminating light‐induced photoelectric artifacts during neuromodulation. We compared the temperature resolution of our tRTD in the tRTD‐MEA with previously reported transparent^[^ ^27^, ^42^ ^]^ and opaque^[^ ^26^, ^32^, ^43^, ^44^, ^45^ ^]^ electrical temperature sensors (Figure 1d). The resolution of our tRTD was 0.063 °C, superior to other transparent electrical temperature sensors. Compared to opaque temperature sensors, our tRTD maintained excellent temperature sensing resolution while directly measuring the photothermal effect. Additionally, our transparent electrode in the tRTD‐MEA exhibited significantly lower baseline noise (1.22 µV_rms_) and lower area‐normalized impedance (0.49 Ω·cm^2^ at 1 kHz) compared to other transparent^[^ ^46^, ^47^, ^48^, ^49^ ^]^ and opaque^[^ ^50^, ^51^, ^52^, ^53^ ^]^ electrodes (Figure 1e).

### 2.2 Electrical, and Optical Characterization of Transparent Temperature Sensor (tRTD) and Direct Sensing of Photothermal‐Induced Heat

**Figure 2:**
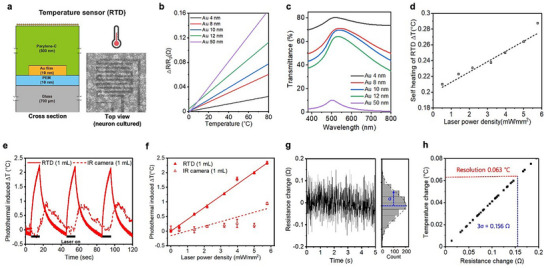
Electrical and optical characteristics of transparent temperature sensor (RTD) and direct sensing of photothermal‐induced heat. a) Schematic illustration of cross‐section and optical top‐view image of the transparent temperature sensor. b) Temperature coefficient of resistance (TCR) and c) optical transparency of ultrathin Au RTD on various thicknesses of Au (ΔR: resistance change, R_0_: initial resistance). d) Characterization of laser‐induced self‐heating of RTD in 1 mL cell culture media (depths: 3.08 mm) environments (ΔT: temperature change). e) Photothermal‐induced temperature changes at the tRTD‐MEA interface, monitored by IR camera and transparent RTDs at a laser power density of 5.77 mW mm^−^^2^ with 3 repeated on/off (10 s/30 s) cycles. Photothermal‐induced temperature changes (ΔT) are calculated based on differences from baseline temperature without laser irradiation. f) Photothermal‐induced temperature changes at the interface in 1 mL of culture media environments according to different laser power densities (0–5.77 mW mm^−^^2^). g) Baseline resistance fluctuation of the temperature sensor at 25 °C constant temperature condition (σ = standard deviation of the resistance fluctuation). h) Sensing resolution of the temperature sensor: Temperature sensing resolution = (3σ/R_o_) ÷ (TCR of the temperature sensor), where R_o_ is the average baseline resistance of the RTD at room temperature.

We integrated 4 sets of transparent ultrathin Au RTDs below the photothermal layer to sense the heat generation (**Figure** **2**a). One of our main strategies is to fabricate tRTDs and electrodes simultaneously with minimized fabrication steps. For fabrication, we sequentially coated positively charged polyethyleneimine (PEI) and negatively charged poly(4‐styrene sulfonic acid) (PSS) three times by spin‐coating on the glass substrate to form a polyelectrolyte multilayer (PEM), followed by the deposition of a 10 nm‐thick Au layer. Compared to deposited Au without PEM coating, pre‐PEM coated Au film shows reduced grain boundaries, forming a smoother and more connected film, indicating better conductivity (Figure S4a, Supporting Information). After a 20‐min heat treatment at 150 °C, the pre‐PEM coated 10 nm Au film exhibits lower sheet resistance (3.77 Ω per sq) than the as‐deposited Au film (6.67 Ω per sq). This confirms that the ultrathin Au film is conductive enough for the RTDs and electrodes (Figure S4b, Supporting Information). The Au(10 nm)/PEM/glass also shows higher transparency than Au(10 nm)/glass, with no noticeable change in transmittance after the heat treatment (150 °C for 20 min) (Figure S4c, Supporting Information).

Next, we optimized the sensitivity of the temperature sensors, defined as temperature coefficient of resistance (TCR), by varying the Au thickness from 4 to 50 nm (Figure 2b). All transparent ultrathin Au RTDs exhibited excellent linearity (r^2^ > 0.99), with TCR ranging from 3.03 × 10⁻⁴ (°C⁻¹) to 2.09 × 10⁻^3^ (°C⁻¹) for 4–50 nm. As the Au film thickness increased, transparency decreased across all wavelengths (380–800 nm) (Figure 2c). Balancing sheet resistance, TCR, and transparency of the Au film, we settled on 10 nm as the optimized thickness, yielding our RTD with a TCR of 9.70 × 10⁻⁴ °C^−1^, sheet resistance of 3.77 Ω per sq, and 71.2% transmittance at 535 nm wavelength. We also assessed the self‐heating of RTDs during NIR laser irradiation. In 1 mL cell culture media, the self‐heating temperature increased by less than 0.3 °C at 5.77 mW mm^−^^2^ NIR laser intensity, which would be insufficiently small for affecting neural activities (Figure 2d).

We further characterized photothermal‐induced temperature changes in air conditions without obstructions, ensuring synchronization between tRTD and IR camera temperature measurements (Figure S5, Supporting Information). The temperature increase due to photothermal effects shows a linear relationship with laser power density in both measurement types, with only a 3.8% difference between tRTD and IR camera at 5.77 mW mm^−^^2^. Subsequently, we simultaneously quantified photothermal heat‐induced temperature changes in 1 mL cell culture media using tRTD and an IR camera (Figure S6a, Supporting Information). Following repeated photothermal modulations (on/off: 10/30 s), tRTD accurately sensed temperature changes synchronously with laser irradiation (Figure 2e). In contrast, IR camera temperature readings show inaccurate magnitude due to the inability to monitor cell interface temperature changes through the culture media. Temperature measurements by the IR camera were up to 64% lower than those by the tRTD across a 0.5–5.77 mW mm^−^^2^ power density range (Figure 2f). Additionally, IR camera readings exhibited nearly a 10‐second delay compared to tRTD readings, indicating the inability of real‐time monitoring using an IR camera (Figure 2e). Similar trends were observed in 2 mL culture media condition during photothermal modulation (Figure S6b, Supporting Information).

To characterize the temperature sensing resolution of the tRTD, we first measured the baseline fluctuation of the tRTD resistance under constant temperature conditions at 25 °C (Figure 2g). The standard deviation (σ) of the tRTD resistance fluctuation was 52 mΩ when the RTD baseline resistance (R_o_) is 2.49 kΩ. We then calculated the temperature sensing resolution (T_res_) of the tRTD by dividing the ratio of 3σ to R_o_ by the TCR calculated from Figure 2b, resulting in a tRTD resolution (T_res_) of 0.063 °C (Figure 2h):

(1) $$\frac{\Delta R}{R}=\left(TCR\right)\;\times \Delta T$$

(2) $$T_{res}=\left(\frac{3\sigma }{R}\right)\;\left(\frac{1}{TCR}\right)$$

where 3σ represents the minimum measurement resolution of the resistance changes of the RTD.

Next, we simulated photothermally generated heat transfer using COMSOL and analyzed resulting temperature changes to validate trends observed with our tRTDs in different environments. Simulation parameters and temperature distributions are detailed in Figure S7a–c and Table S1 (Supporting Information). Simulation results confirmed temperature consistency between the top surface of the 500‐nm thick parylene‐C passivation layer, where the photothermal layer is located, and the area directly under it where tRTDs are positioned (Figure S7d, Supporting Information). Therefore, tRTD‐monitored temperatures accurately reflect photothermal layer surface temperatures interfacing with neuronal cells. Simulation results mirrored experimental findings, with ≈ 85% reduction in peak temperature changes when 1 mL of cell culture medium was introduced, owing to its higher heat capacity compared to air (Figure S7e; Table S1, Supporting Information).

### 2.3 Electrochemical and Optical Characteristics of Transparent Electrodes

**Figure 3:**
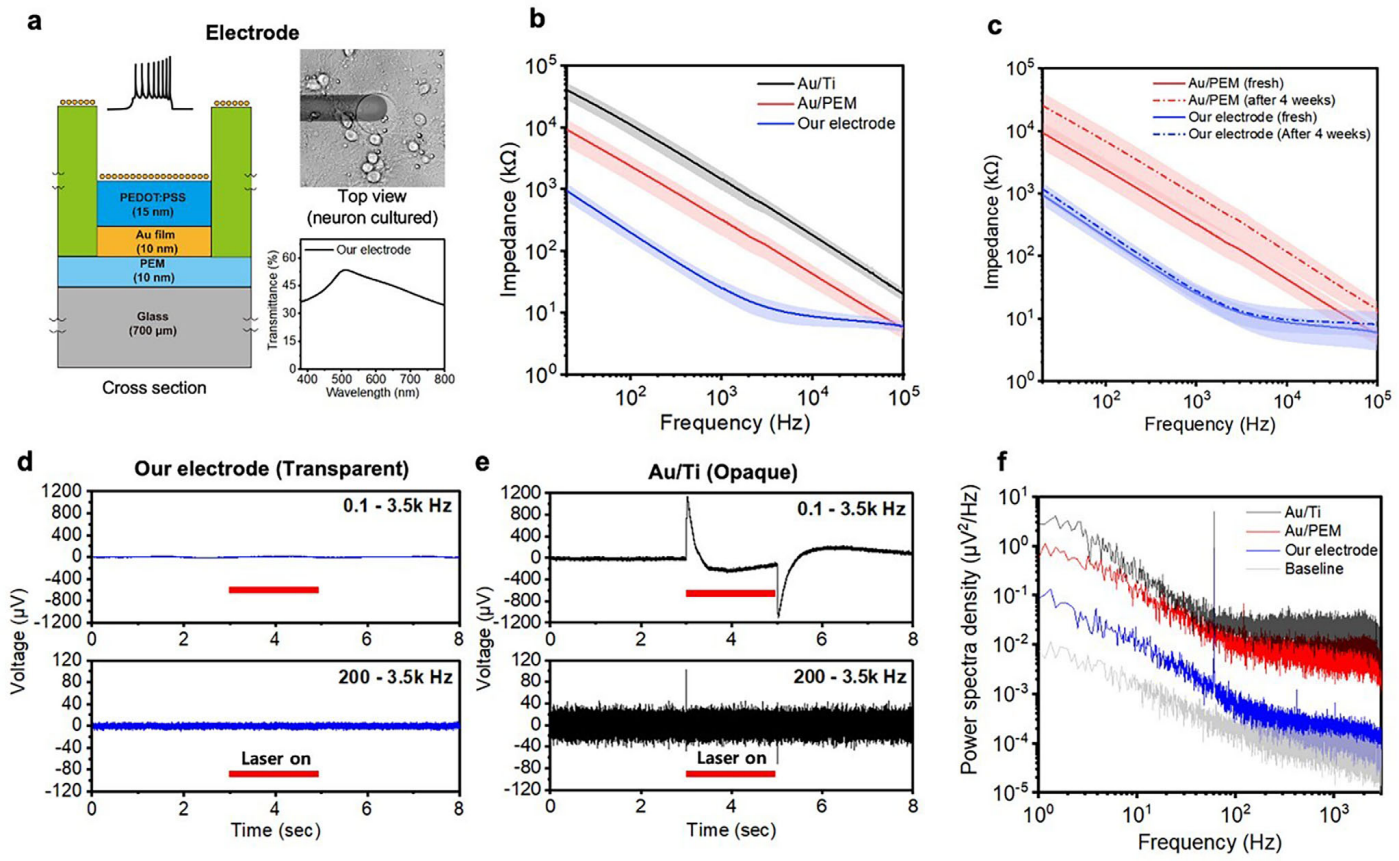
Electrochemical and optical characteristics of transparent electrodes. a) Schematic illustration of cross‐section, top‐view image, and transmittance spectrum of transparent electrodes. b) The magnitude of Bode plots showing representative EIS data for three different types of electrodes on glass substrates. *Au/Ti*: Au(50 nm)/Ti (5 nm), *Au/PEM*: Au(4 nm)/Au(10 nm)/Polyelectrolyte multilayer (PEM), *Our electrode*: Au(4 nm)/PEDOT(15 nm)/Au(10 nm)/PEM. Error bars represent standard deviation (*Au/Ti*: n = 4, *Au/PEM*: n = 21, *Our electrode*: n = 16). c) Long‐term stability of transparent electrodes in aqueous solution. Each electrode was immersed in DI water for 4 weeks at 37.5 °C. Error bars represent standard deviation (*Au/PEM*: n = 21, *Our electrode*: n = 16). d, e) Light‐induced photoelectric artifacts of our transparent electrodes and opaque Au/Ti electrodes in PBS solution during 2‐s long NIR laser irradiation (785 nm) with different bandpass filter conditions. f) Power spectral density of recorded electrode baseline noise (1/f in lower frequency range and thermal noise in higher frequency range).

**Figure** **3**a depicts the cross‐section scheme of electrode and top view image of electrode with hippocampal neurons cultured above it. Our sandwich‐structured Au(4 nm)/PEDOT(15 nm)/Au(10 nm)/PEM electrode exhibited an Au color and 53.5% transparency at 520 nm. Furthermore, our 50‐µm diameter transparent electrode demonstrated an average impedance of 25.2 kΩ at 1 kHz, which is 58‐ and 9.2‐times lower impedance than *Au/Ti*, Au(50 nm)/Ti(5 nm), and *Au/PEM*, Au(4 nm)/Au(10 nm)/PEM, electrodes at 1 kHz, respectively (Figure 3b; Figure S8a, Supporting Information). The phase angle of the transparent electrode exhibited the least capacitive behavior among all structures (Figure S8b, Supporting Information). One reason for the improvement in impedance could be the enhanced surface roughness (R_q_), providing a more effective surface area due to the complex structure of the PEM seed layer under the ultrathin Au and the 4‐nm Au island on top of the ultrathin Au.^[^ ^50^ ^]^ Compared to the typical opaque electrode (*Au/Ti*, R_q_: 1.044 nm), the ultrathin Au (*Au/PEM*) exhibited a larger surface roughness of 2.522 nm, resulting in enhanced double‐layer capacitance (C_dl_), calculated from Nyquist plots and equivalent circuit model (Figures S8c and S9; Table S2, Supporting Information). Our transparent electrode (*Our electrode)* not only displayed the highest surface roughness (12.301 nm), enhancing the effective surface area, but also included transparent PEDOT:PSS, known for its good charge transfer characteristics.^[^ ^54^ ^]^ Therefore, spin‐coated PEDOT:PSS and the top‐located 4‐nm Au surface contributed to reduced electrochemical impedance and more efficient charge transfer (Figure S10; Table S2, Supporting Information). Additionally, *our electrode* exhibited long‐term aqueous stability, showing only an 8% increase in impedance at 1 kHz after immersion in an aqueous solution for 4 weeks (Figure 3c). Despite using Au, our transparent electrode shows no significant photoelectric artifact noise, enabling accurate and stable measurement of neural spike signals during photothermal stimulation due to its reasonable optical transparency (Figure 3d). In contrast, *Au/Ti* electrode exhibited a photoelectric artifact of ∼90 µV even after 200–3.5 kHz band‐pass filtering, leading to false positive spike detection (Figure 3e). Lastly, our transparent electrode shows minimal power spectral density (PSD) of recorded electrode baseline noise compared to other electrodes, closely resembling the baseline noise of the measurement system (Figure 3f).

### 2.4 Analysis of Photothermal Neuromodulation on tRTD‐MEA

**Figure 4:**
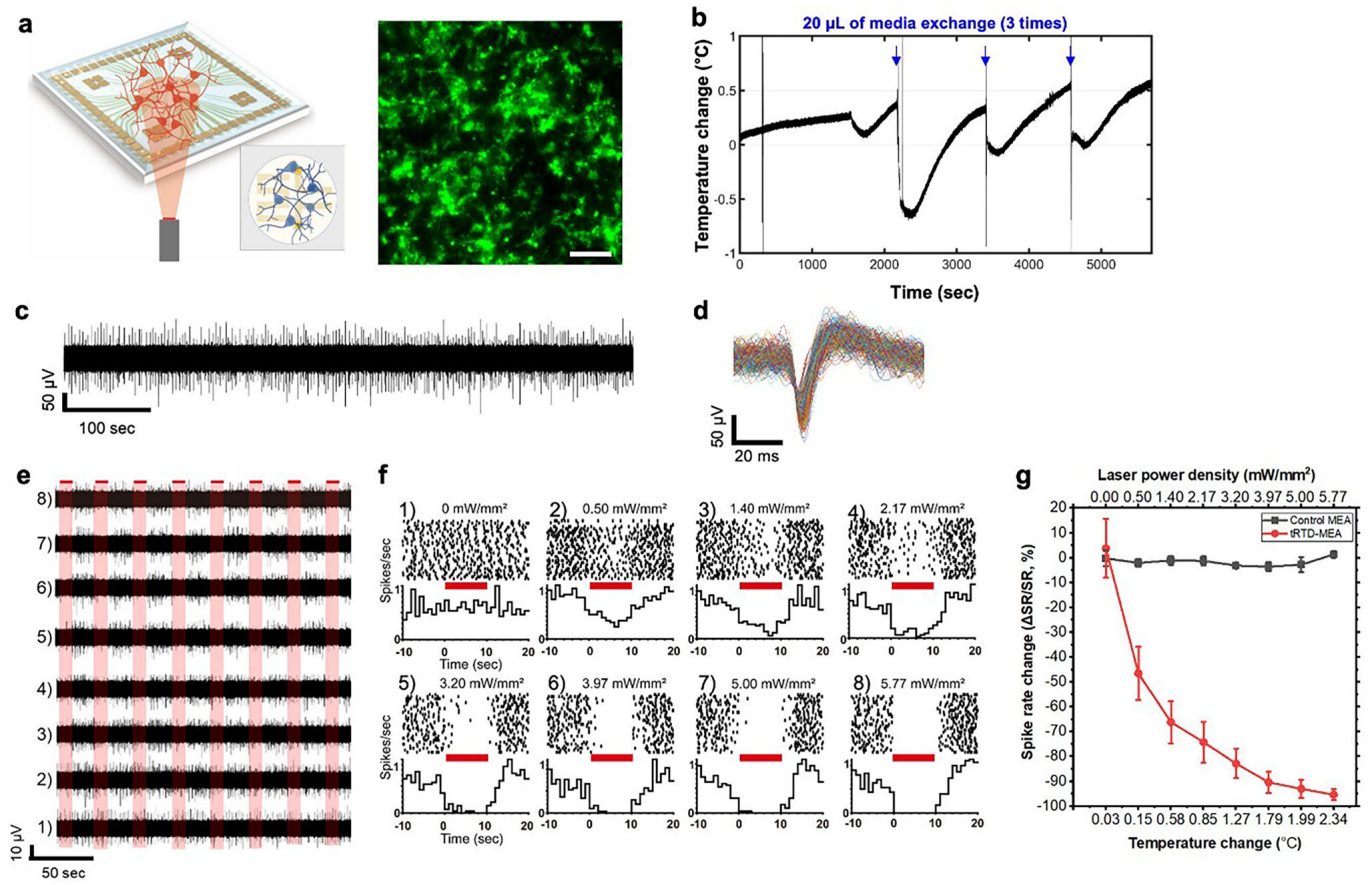
Analysis of photothermal neural inhibition on tRTD‐MEA. a) (Left) Schematic illustration of the photothermal neural inhibition experimental setup with hippocampal neurons cultured on tRTD‐MEA. (Right) Fluorescence image of the cultured hippocampal neurons on tRTD‐MEA (scale bar = 100 µm). b) Precise detection of small temperature changes during the exchange of 20 µL of media out of 1 mL of cell culture media. c) A representative neural spike activity trace on tRTD‐MEA. d) Accumulated spike waveform from (c). e) Electrical recording traces of tRTD‐MEA during photothermal stimulation. Red bars represent 10‐s long NIR laser irradiation periods (NIR laser power density: 0–5.77 mW mm^−2^). f) Peri‐stimulus time histogram (PSTH) and raster plots of photothermal neural inhibition experiments according to the results of (e). g) Quantification of spike rate changes on tRTD‐MEA during the NIR photothermal stimulation (ΔSR: spike count change, spike count during the light stimulation – mean value of spike count before and after the light stimulation; SR = mean value of spike count before and after the light stimulation). The x‐axis (top) of the graph represents laser power density, and the other x‐axis (bottom) represents temperature changes according to the NIR intensity (n = 6).

Through the tRTD‐MEA chip, we demonstrated whether the photothermal stimulation could inhibit the neural spike activity. **Figure** **4**a shows the scheme of photothermal stimulation‐induced inhibition of the hippocampal neural networks and a fluorescence image of cultured hippocampal neurons on the tRTD‐MEA. With the high‐resolution temperature sensing capability of the tRTD‐MEA, we can monitor changes in the cellular environment temperature even when a small volume of 20 µL of cell media is added to 1 mL of cell media, as shown in Figure 4b. Even with a 2% of added volume, the temperature sensing in our system is sensitive enough to detect a transient temperature decrease of 0.5–1.5 °C and continuously monitor a recovery to the baseline temperature in 5–10 min (Figure 4b). These findings will be helpful for studies that analyze the effect of chemicals or drugs on biological behaviors while excluding the effect of temperature changes.

Before we performed neural recording and photothermal stimulation experiments, we verified that the fabricated tRTD‐MEA had no cytotoxicity and could be used for neuronal cell experiments. To confirm the biocompatibility of the cells, we cultured hippocampal neurons from E18 Sprague‐Dawley rats on commercial MEAs (as control MEAs) and tRTD‐MEAs with excellent imaging quality through the transparent sensors (Figure S11a, Supporting Information). We performed a live/dead assay of hippocampal neurons cultured in control MEAs and tRTD‐MEAs. Figure S11b (Supporting Information) shows fluorescence images of hippocampal neurons on the control MEA and tRTD‐MEA. The viability of the control MEA was 51.70 ± 0.96%, and the tRTD‐MEA was 51.07 ± 2.24% (Figure S11c, Supporting Information), showing no significant difference (p = 0.4145, unpaired one‐tailed t‐test). These results indicate that tRTD‐MEA is as suitable for cell experiments as the control MEA.

Photothermal stimulation for neural signal inhibition was performed between 14 and 21 days in vitro (DIV). We confirmed that the hippocampal neurons cultured on the tRTD‐MEA grown normally and generated neural signals (Figure 4c), and the spike waveforms were normal and continuous (Figure 4d). Figure S12a (Supporting Information) shows a neural spike activity trace for different NIR irradiation durations with 6.41 mW mm^−2^ laser power density and spike waveform before and after photothermal stimulation. The raw data trace shows a clear inhibition of neural signals during photothermal stimulation. The range of the laser power density for the inhibition is lower than the previously reported photothermal inhibition experiments^1,6^. Following photothermal stimulation, neuron activity remained sustained, showing no significant alterations in spike waveform and amplitude (Figure S12b, Supporting Information). The life span of neurons in the experimental group that performed the photothermal stimulation experiment was not significantly different from that in the control group (data not shown).

We investigated the correlation between temperature change and neural signal inhibition during the photothermal stimulation by using the tRTD‐MEA. The neural activities of neurons decreased proportionally as the laser power density increased from 0 to 5.77 mW mm^−2^, indexed from 1 to 8 in Figure 4e, showing a clear inhibitory stimulation effect. This gradual decrease in neural activity aligns the experimental observations reported in previously studies.^[^ ^1^, ^4^, ^6^, ^8^ ^]^ Figure 4f shows perievent raster and poststimulus time histogram (PSTH) from cultured hippocampal neurons on the tRTD‐MEA with different laser power intensities during 30 times of repeated stimulation, respectively. For the NIR stimulation conditions (0 to 5.77 mW mm^−2^), the temperature increase was measured by the tRTD‐MEA, and the results ranged from 0.03 to 2.34 °C. The hippocampal neurons grown on the tRTD‐MEA shows 95.35% inhibition (i.e., spike rate (SR) change ratio, ΔSR/SR) at a local temperature increase of 2.34 °C (Figure 4g). Control MEAs without any photothermal layer showed no neural inhibition despite the same power of NIR illumination.

This study observed a similar inhibition of neural activity at 5.77 mW mm^−2^ despite the difference in the NIR laser power range applied to the hippocampal neurons used in the previous studies (15–21 mW mm^−2^).^[^ ^1^, ^6^, ^8^ ^]^ The temperature change that led to neural inhibition was ≈ 2.3 °C, which was measured on the surface of the cells rather than the upper layer of the cell culture medium. This suggests that we more accurately measured the heat transmitted to the cell surface with the tRTD‐MEA and applied the minimum amount of photothermal stimulation necessary to inhibit neural signals. These results are an important finding in that they can provide a more general standard for photothermal neural modulation. Temperature change is a simpler and more intuitive indicator to understand than the light sources' intensity or the applied thermal energy. It can unify the various light sources used in existing stimulation experiments using light, and the experimental conditions are presented differently for different target cells, tissues, and organisms.

## 3 Discussion

High‐precision modulation of neural electrophysiology is important for understanding neural communication intricacies and for diagnosing and treating neural disorders. Photothermal neuromodulation presents a remote and non‐genetic approach to neural modulation characterized by high spatiotemporal resolution. Despite these advantages, there has been a lack of research that clearly shows a correlation with neuronal activity based on temperature changes. In this work, we claim a correlation between temperature changes and an inhibition phenomenon of neuronal activity by fabricated tRTD‐MEA. Until now, it has been argued that the temperature increases in photothermal stimulation result in excitation by the temperature‐sensitive TRP ion channels and inhibition by TREK channels.^[^ ^1^, ^17^ ^]^ However, it depends on the different irradiation conditions, such as different light sources and how abruptly the temperature increases. Even if there was a temperature measurement, it was not exactly the temperature of the photothermal layer in contact with the cells.

Here, owing to the capability of our tRTD‐MEA platform, we attempted to confirm the clear result by measuring the temperature close to the cell membrane. As a result, the temperature increased by ≈2.3 °C upon 10 s of NIR illumination with 5.77 mW mm^−2^. This led to over 95% in average neural inhibition of the in vitro hippocampal neural network primarily cultured from a rat embryo. In comparison, it has been reported that the temperature increases of 0.2–2.0 °C upon visible light illumination could lead to the suppressed spiking in multiple brain regions of wild‐type opsin‐independent mice in vivo.^[^ ^55^ ^]^ Using our platform, we were able to directly measure a gradual temperature increase of 2.34 °C over 10 s through photothermal‐induced heating at a laser power density of 5.77 mW mm^−^^2^. During the stimulation, the maximum instant temperature increases per unit time, the thermal heating rate, was ≈2 °C s^−1^ when the laser is just turned on, and the heating rate saturated to 0.2–0.5 °C s^−1^. On the other hand, pulsed laser based infrared neural stimulation is analyzed to be causing 30–45 °C s^−1^ (0.30–0.45 °C increase for 10 m s),^[^ ^56^ ^]^ or even higher (4 °C increase for 10 m s),^[^ ^57^ ^]^ which is orders of magnitude higher than the photothermal stimulation condition in our work. These results support the need for accurate measurements closer to the surface of the cells. The tRTD‐MEA will be useful for temperature‐based stimulation of neuronal activity, especially for in‐depth investigation of related temperature‐sensitive ion channel responses, leading to either excitation or inhibition. Given the previously challenging technology to quantitatively analyze the photothermal heating rate in the field of neural stimulation, our platform can become useful in deepening the understanding of this heat induced neural modulation research field.

We believe that the neural modulation results observed from a 2.3 °C temperature change in our in vitro study represent a reasonable value for neuromodulation, comparable to other light‐based neural stimulation studies. In an in vivo infrared neural stimulation (INS) study using rats, cortical neurons were stimulated with an optrode at 10.5 mW for 2 min using 1550 nm wavelength light, resulting in an ≈5 °C temperature increase and a decrease in the normalized firing rate from 1 to 0.5.^[^ ^28^ ^]^ Despite using different thermal methods, the range of temperature increase reported in these works is similar to the results observed in our photothermal neural stimulation experiment. Through these results, we were able to quantitatively confirm the correlation of photothermally induced temperature changes with various previously reported neural activity modulation results.

Ultimately, this change in temperature affects the activity of neurons in the brain because, as mentioned in the introduction, the thermosensitive properties of the cells are affected and acted upon. Therefore, the development of such a transparent multifunctional platform is of great significance in that it allows us to obtain important information. Although our platform is based on simple principles and processes, it is fabricated using high‐resolution microfabrication, allowing us to fabricate temperature sensor resolution as small as a few µm. Further development of this technology will enable the measurement of temperature changes in more localized areas down to the µm scale (single cell level), as well as the deployment of highly integrated temperature sensors, which will allow for deeper characterization of neural networks and changes in cellular and intercellular properties in response to different temperature changes.

Moreover, there is a growing interest in monitoring temperature changes resulting from biological mechanisms not only in neural stimulation but also in various biochip platforms, such as drug screening or cell biology. We believe that the tRTD‐MEA will enable more in‐depth research in this area, allowing for simultaneous analysis of the biological mechanisms of injected drugs and temperature changes, thereby distinguishing their respective influences. For example, we can simultaneously analyze the biological mechanisms of injected drugs and the biological mechanisms of temperature changes and distinguish their influence. As an example, it has been reported that the ion concentration change by injection of an additional small volume of media in the cell environment could induce a significant cell temperature change, which may have to do with the mechanism of temperature‐sensitive ion channels.^[^ ^58^ ^]^ With the high‐resolution temperature monitoring capability of our tRTD‐MEA, we can monitor these changes in the cellular environment temperature even when a small volume is exchanged as shown in Figure 4b. These findings will be helpful for studies that analyze the effect of chemicals on biological behaviors while excluding the effect of temperature changes.

## 4 Conclusion

In this study, we demonstrated the capability to directly sense photothermal‐induced temperature changes at the neuronal cell interface during photothermal neuromodulation. By integrating transparent resistive temperature detectors (RTD) with a microelectrode array (MEA) and photothermal layer into a multifunctional transparent RTD‐MEA, we monitored neural activity, applied photothermal stimulation, and precisely measured the resulting temperature changes near the cell surface in real‐time. Our main finding is the correlation that an average 2.34 °C temperature increase at the neuron‐material interface resulted in over 95% inhibition of hippocampal neural activities. The ability to quantitatively correlate specific temperature elevations with modulation of neural firing provides a new quantitative standard for understanding the mechanisms of photothermal neuromodulation. This platform overcomes the limitations of previous methods that indirectly measured and estimated temperature changes. It enables a much deeper investigation of temperature‐sensitive ion channel responses and their implications in various neurological disease models and treatments.

## 5 Experimental Section

### Device Fabrication

For fabrication of the tRTD‐MEA, each of 0.15 wt.% of PEI (Sigma‐Aldrich product 181978, 50 wt % sol. In H_2_O) and 10 mg mL^−^ of PSS (Sigma‐Aldrich, product 561223, 18 wt.% sol. in H_2_O) were dissolved in 10 mm NaCl aqueous solutions and then layer‐by‐layer spin‐coated on the acetone/IPA cleaned 700 µm thickness of quartz glass substrate at 2500 rpm. While coating each layer, DI water was spin‐coated at 2500 rpm to prevent the agglomeration of each PEI and PSS layer. Afterward, the negative photoresist (Sigma‐Aldrich, AZ nLOF) was spin‐coated at 3500 rpm, followed by soft‐baking at 110 °C for 1 min. Next, UV exposure (365 nm wavelength) was performed at 70 mJ cm^−2^ exposure energy, followed by post‐exposure baking at 110 °C for 1 min. Subsequent to that, a photoresist was developed for 90 s with a photoresist developer (Sigma‐Aldrich, AZ‐300), followed by rinsing in DI water. 10 nm of Au was deposited by a thermal evaporator (Korea Vacuum Tech, South Korea) at 0.1 Å s^−1^ of deposition rate at 3–5 ×10^−6^ (Torr) of chamber pressure to fabricate tRTD‐MEA. Au film was thermally treated at 150 °C for 20 min to lower the sheet resistance.

500 nm thickness of parylene‐C was deposited by a parylene coating system (Nuritech, NRPC‐500, South Korea) with pre‐coating of adhesion promoter (Sigma‐Aldrich, Silane A174). Afterward, negative photoresist (Sigma‐Aldrich, AZ nLOF) was patterned as an etch mask followed by oxygen plasma reactive on etching (Fabstar, TTL, South Korea) for 3.5 min at 150 W power with 40 sccm oxygen flow.

Next, PEDOT:PSS mixture solution, PEDOT:PSS (Heraeus, PH1000): DI = 1:2 with 0.2 wt.% of GOPS (3‐ glycidyloxypropyl)trimethoxysilane, was spin‐coated at 1000 pm followed by thermal treatment at 110 °C for 1 min to crosslink GOPS added PEDOT:PSS layers. Then, the negative photoresist was lifted‐off to pattern the PEDOT:PSS‐coated electrode opening layers. Next, 4‐nm of Au was deposited by a thermal evaporator (Korea Vacuum Tech, South Korea) at 0.1 Å s^−1^ of very low deposition rate at 3–5 ×10^−6^ (Torr) of chamber pressure followed by the heat treatment at 150 °C for 20 min to completely dewet the 4‐nm of deposited Au layer on parylene‐C film function as photothermal induced heat generation. Afterward, a 50 nm thickness of Au was deposited by a thermal evaporator (Korea Vacuum Tech, South Korea) at 0.5 Å s^−1^ deposition rate at 3–5 ×10^−6^ (Torr) of chamber pressure at a contact pad with shadow masking. Last, glass ring (Inner diameter: 20.32 mm) was attached the center of the tRTD‐MEA by Polydimethylsiloxane (PDMS) (Sigma Aldrich, Sylgard 184):Silicon elastomer curing agent (Sigma Aldrich, Sylgard 184 10 g clip‐pack Curing agent) = 10:1 of mixture followed by thermal crosslinking in the oven at 60 °C for 1 h.

### Morphology Characterization

The surface of the electrode and 4‐nm Au island of photothermal layers were characterized with FE‐SEM (Hitachi/SU8230, Acceleration voltage: 1.6 kV, Working distance:5.4‐6 mm), atomic force microscopy (AFM) (Park Systems/XE‐150, Scanning area: 1 µm × 1 µm, 256‐bit × 256‐bit, Scanning speed: 1 Hz), transmission electron microscopy (TEM) (Thermoscientific/Themis Z, Acceleration voltage: 300 kV, Resolution: 60 pm) and stylus profiler (Bruker, Dektak XT, Stylus force: 3 mg).

### Electrical, Optical Characterization

The sheet resistance of Au film was characterized by a four‐point probe system (AIT/CMT‐SR2000N). The resistance of tRTD‐MEA and interconnect Au lines was characterized by a two‐point probe system (Model 8000, MS Tech, South Korea). The TCR of various thickness RTDs were characterized by a four‐point probe system (Model 8000, MS Tech, South Korea) with carefully monitored resistance change during temperature increase. The light‐induced self‐heating of RTD was characterized with RTD sensing module (National Instruments, NI 9229) during 785 nm near‐infrared CW laser (450 mW, TEM‐F‐785‐450 mW, CNI Tech., Changchun, China) irradiation below RTD at 30 mm distance at 0.5–5.77 mW mm^−2^ power density range for 60 s each of in air and aqueous media conditions. The transparency of tRTD‐MEA was acquired by UV−vis‐NIR optical spectrometer (Cary 5000, Agilent Technologies, USA).

### Electrochemical Characterization

The impedance and phase angle of electrodes were characterized by an LCR meter (NF Model ZM2410, NF corporation) using a 10‐mV sine wave signal with a range of frequency from 20 to 100 kHz. All the electrodes were covered with Dulbecco's Phosphate Buffered Saline (DPBS) solution (Sigma‐Aldrich, 806552) to form electrode/electrolyte interfaces. EIS data fitting based on Randles equivalent circuit was performed using a free EIS spectrum analysis software (EISSA, Belarus).

### Electrode Noise and Photoelectric Artifact Characterization

The root mean square noise of the electrodes was characterized with a commercial multichannel in vitro recording system (MEA2100‐Mini‐Sytem, Multi Channel Systems MCS GmbH) with a 60‐channel head stage amplifier (MEA2100‐Mini‐HS60) in an incubator with PBS solution filled in a glass ring of tRTD‐MEA. Light‐induced photoelectric artifacts were also measured by the commercial multichannel in vitro recording system, with 30 mm below placed 785 nm near‐infrared CW laser with 8.3 mm irradiated diameter. Laser modulation was performed at the 5.77 mW mm^−2^ power density with repeated five cycles (on/off: 2 s/5 s) of pulsed square shape waves controlled by a TTL pulse system. The recorded photoelectric artifact signals were filtered by a 200 Hz high‐pass filter in multichannel experimenter software.

### Measurement Temperature Change during a Small Volume of Cell Media Exchange

The temperature change was measured when exchanging media with the tRTD‐MEA. 1 mL of cell culture media was put in the tRTD‐MEA and covered the lid. We then placed the media to be exchanged with the chip, pipette, and pipette tips in a humidified incubator with 5% CO_2_ and 37 °C and waited for at least 2 h for the temperature to equilibrate. We then exchanged 20 µL of media in the incubator and repeated the process 3 times, measuring the temperature, until it equilibrated again. The temperature was measured with the tRTD‐MEA chip in real‐time.

### Photothermal Effect‐Induced Heat Characterization

The photothermal‐induced heat was characterized with the RTD and 4‐point temperature readout module (National Instruments, NI 9226, Excitation current: 100 µA) during 30 mm below placed NIR laser modulation at 0.5–5.77 mW mm^−2^ power density range with repeated three cycles (on/off: 10 s/30 s) of pulsed square shape waves each of in air and aqueous media condition. Simultaneously, the surface temperature of the RTDs was monitored with an IR camera (A325SC, FLIR) with a close‐up IR lens (T197201, FLIR) upon NIR laser irradiation.

### Cell Culture

A pregnant Sprague‐Dawley rat (Hana‐biotech, Korea) was sacrificed with 100% CO_2_, and embryos (embryonic stage E18 days) were collected from the uterus and stripped of the meninges in an ice‐cold Hank's buffer salt solution (HBSS; Welgene, Korea), then the hippocampus regions were dissected under a microscope. The hippocampal neurons were dissociated by gentle pipetting preventing bubbles from forming. The cells were centrifuged for 2 min at 1000 rpm, and the supernatant was discarded with cell culture plating medium. The pellet was resuspended with plating medium consisting of serum‐free Neurobasal medium (Gibco), supplemented with B27 (Gibco), 2 mm of GlutaMAX (Gibco), 12.5 µM of glutamate (Sigma) and penicillin‐streptomycin (Gibco). Using a cell strainer (BD Falcon), the cell suspension was filtered and collected. Then neural cells were calculated and plated at the density of 1000 cells mm^−2^. After 3 DIV (days in vitro), half of the plating medium was replaced with a maintenance medium that did not contain glutamate. The maintenance medium was replaced regularly every 4 or 5 days. The cell culture was maintained in a humidified incubator with 5% CO_2_ and 37 °C. All in vitro experiments were performed in accordance with the guidance of the Institutional Animal Care and Use Committee (IACUC) of Daegu Gyeongbuk Institute of Science and Technology (DGIST), and all experimental protocols were approved by the IACUC of DGIST (DGIST‐IACUC‐21041903‐0002).

### Optical Imaging and Cell Viability Test

All phase‐contrast and fluorescence images of cultured hippocampal neurons were obtained using a CMOS camera (DS‐Qi2, Nikon) mounted on an inverted microscope (ECLIPSE Ti, Nikon) with 10×, 20×, and 40× objective lens. A live/DEAD viability/cytotoxicity kit for mammalian cells (Invitrogen) was used to measure the viability of cultured hippocampal cells. Performed the biocompatibility test between 14–21 DIV cultured neurons on the tRTD‐MEA and control MEA. To conduct the live/dead assay, 200 µL of staining solution (consisting of 2 µM calcein AM and 4 µM ethidium homodimer‐1 in DPBS) was introduced to cultured cells along with 800 µL of culture medium. The mixture was then incubated for 20 min. The fluorescence signals from the live/dead assays, utilizing the typical excitation/emission wavelengths (494/517 nm for live cells and 528/617 nm for dead cells), were effectively measured. All procedures counted dead cells by applying neuron nucleus size using ImageJ. The viability of neurons cultured with Control and RTD MEAs was analyzed by unpaired *t*‐test (n = 4 ROIs from 3 chips each). The dead cells in both MEAs were mostly those that died during the seeding process in the primary culture, while the growing cells attached to the tRTD‐MEAs grew as well as the control MEA. The viability results were tested statistically using an unpaired one‐tailed t‐test. There was no significant difference between samples, and the P value was 0.4145.

### In Vitro Neural Cell Recording

All neural activity recording experiments were performed from 14 to 21 DIV cultured samples. Electrophysiological signals from cultured hippocampal neurons were sensed by electrodes of MEA. The sensed signals were amplified and digitized with a 60‐channel head stage amplifier (MEA2100‐Mini‐HS60, Multi‐Channel Systems MCS GmbH; 24‐bit, sampling frequency: 25 kHz) and a commercial data acquisition system (MCS‐SCU‐in‐vitro, Multi‐Channel Systems, Germany). A reference electrode integrated into the MEA was connected to the amplifier ground. To detect spikes from extracellular neural signals, the raw data stream was filtered by a digital 2^nd^‐order Butterworth high pass filter (f_c_ = 200 Hz), and the spike detection threshold was set at five standard deviations of the background noise level. Neural recording and spike detection were performed using a Multichannel Experimenter (Multi Channel Systems, Germany), and spike train analysis was performed with a NeuroExplorer (Nex Technologies, USA).

### Photothermal Neuromodulation

A near‐infrared diode laser at 785 nm (TEM‐F‐785, CNI lasers) was utilized as a light stimulation source. The illumination area of the laser beam formed 8.3 mm in diameter on the control MEAs and tRTD‐MEAs (Figure 2b). Cultured hippocampal neurons on both MEAs were irradiated with the 785 nm wavelength NIR laser. The stimulated light intensity was 0, 0.50, 1.40, 2.17, 3.20, 3.97, 5.00, and 5.77 mW mm^−2^. One cycle was performed by turning off the laser for 20 s followed by turning on 10 s. This cycle was repeated for 30 times. To ensure clear evidence of signal suppression, a minimum photothermal stimulation duration of 10 s was employed for statistical analysis. If the stimulation period is set too short, there is a risk of misinterpreting the absence of signals as a result of weak neuronal activity unrelated to the cultured neurons or external photothermal stimulation. To avoid such misinterpretations, a baseline stimulation duration of at least 10 s was established for the experiments.

### Data Analysis of Photothermal Inhibition

To assess the degree of neural activity suppression, attention was directed toward active channels exhibiting an average firing rate surpassing 0.1 spikes per second. Subsequently, the spike rate change was computed using the following Equation (3):

(3) $$Spike\;rate\;change\;\;\left(\%\right)=\frac{\Delta SR}{SR}\;\times 100$$

where SR: mean value of spike count before and after the light stimulation, ΔSR: spike count during the light stimulation – mean value of spike count before and after the light stimulation.

### Finite Element Method Analysis of Photothermal Stimulation

Multiphysics v5.5a (COMSOL Inc., Burlington, MA, USA) was used conduct physical simulation of the photothermal stimulation. To maximize the accuracy of the simulation, we set up the specification of a microelectrode array system similar to the experiment and applied periodic heat sources to the Au layer (Figure S7a,b; Table S1, Supporting Information). The heat transfer simulation was performed based on the following Equation (4):

(4) $$\rho C_{\rho }\frac{\partial T}{\partial t}+\rho C_{\rho }u\cdot \nabla T=Q+k\nabla^{2}T$$

where ρ*, C*_ρ_ *, T(x, y), u, k, and Q(T, x, y)* are mass density (kg/m^3^), heat capacity (J/kg · K), temperature (°C), fluid velocity vector, thermal conductivity (W/m · K), and heat source per volume (W/m^3^), respectively. To analyze the influence of exposed area from the external environment, we further applied heat convection to the surface with the following Equation (5):

(5) $$q_{0}=h\left(T_{ext}-T\right)$$

where h and T_ext_ are the heat transfer coefficient and external temperature, respectively. We set the heat transfer coefficient to 5 W/(m^2^ · K) and external temperature to 28 °C. Material properties for thermal stimulation used in COMSOL can be referred to Table S1 (Supporting Information).

## Conflict of Interest

The authors declare no conflict of interest.

## Author Contributions

D.K., J.W.L., and S.K. contributed equally to this work. D.K., J.W.L., S.K., and H.K. conceived and designed the project. D.K. and S.K. fabricated and characterized the tRTD‐MEA. J.W.L. performed all in vitro experiments neural recording, photothermal stimulation, W.H. and H.K. performed and analyzed the computational thermal simulation. D.K. and J.L. analyzed the electrode noise data. H.K., H.K., and J.J. provided resources for experiments. D.K., J.W.L., H.K., and L.L. analyzed the experimental data. D.K., J.W.L., S.K., W.H., and H.K. wrote the initial draft of the manuscript. The final manuscript was provided with inputs from all the authors; H.K., and L.L. supervised the project.

## Supporting information



Supporting Information

## Data Availability

The data that support the findings of this study are available from the corresponding author upon reasonable request.
