# Supplementary material for: Simultaneous Detection of Neural Activity and Temperature in Photothermal Neural Stimulation
Source: Adv Sci (Weinh). 2025 Mar 26;12(19):2411725. doi: 10.1002/advs.202411725 (PMC12097117; doi:10.1002/advs.202411725)
Supplement: Supplementary file 1 — Supporting Information [file ADVS-12-2411725-s001.docx]

**[Supplementary Data]**

**Simultaneous Detection of Neural Activity and Temperature
in Photothermal Neural Stimulation**

*Duhee Kim^1⸹^, Jee Woong Lee^1.4⸹^*, *Seoyoung Kang^2⸹^, Woongki Hong^1^, Jungha Lee^1^, Hyuk-Jun Kwon^1^, Jae Eun Jang^1^, Luke P. Lee^3,4,5^, and Hongki Kang^1,6,7,8,9^*

^1^ Department of Electrical Engineering and Computer Science, Daegu Gyeongbuk Institute of Science and Technology (DGIST), Daegu 42988, Republic of Korea

^2^ School of Undergraduate Studies, College of Transdisciplinary Studies, Daegu Gyeongbuk Institute of Science and Technology (DGIST), Daegu 42988, Republic of Korea

^3^ Renal Division and Division of Engineering in Medicine, Department of Medicine, Brigham and Women's Hospital, Harvard Medical School, Boston, MA, 02115, USA

^4^ Department of Bioengineering, Department of Electrical Engineering and Computer Sciences, University of California at Berkeley, Berkeley, CA, 94720, USA

^5^ Institute of Quantum Biophysics, Department of Biophysics, Sungkyunkwan University, Suwon, 16419, South Korea

^6^ Department of Biomedical Engineering, Seoul National University College of Medicine, Seoul, 03080, Republic of Korea

^7^ Interdisciplinary Program in Bioengineering, College of Engineering, Seoul National University, Seoul, 08826, Republic of Korea

^8^ Seoul National University Hospital, Seoul, 03080, Republic of Korea

^9^ Institute of Medical and Biological Engineering, Medical Research Center, Seoul National University, Seoul, 03080, Republic of Korea

*Correspondence to: hongki.kang@snu.ac.kr

*^⸹^*: These authors contributed equally.

Keywords: Photothermal neuromodulation, Transparent ultrathin Au, High-resolution direct temperature sensing, Low-noise neural electrodes, Multifunctional microelectrode array


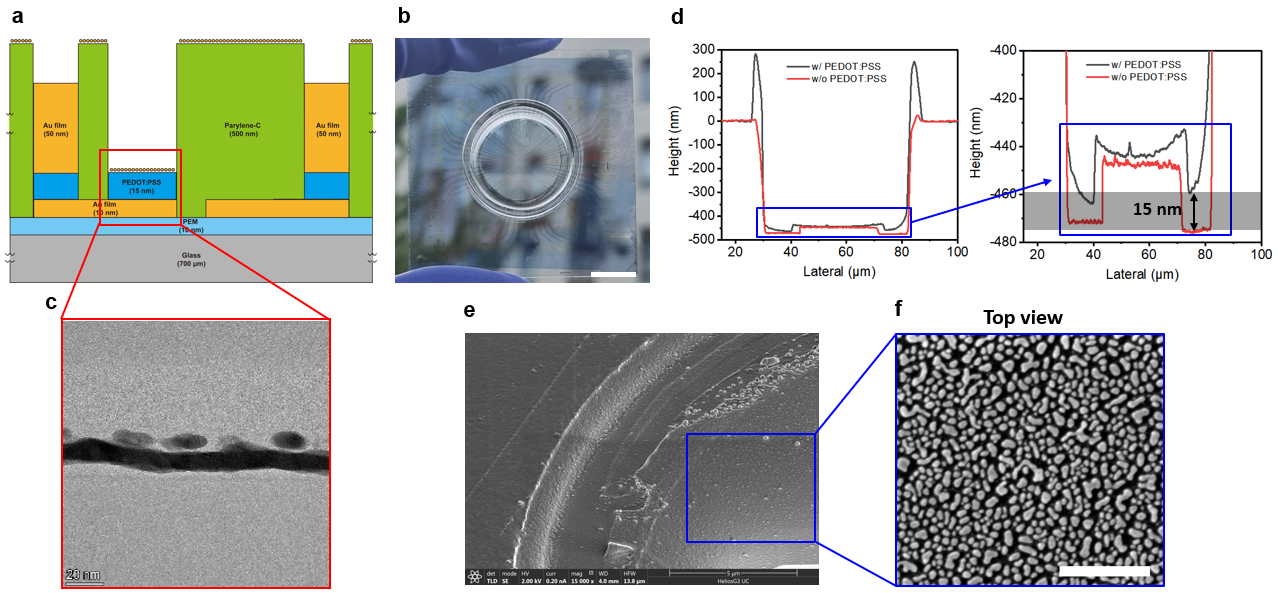


**Supplementary Figure 1. Device structures of transparent RTD-MEA. (a)** Cross sectional schematic of tRTD-MEA and **(b)** digital camera image (Scale bar = 1 cm). **(c)** Cross sectional TEM image of transparent electrode. **(d)** Surface morphologies of transparent electrode (Red line: Au 10 nm/PEM, Black line: PEDOT:PSS/Au 10 nm/PEM). **(e)** Tilted SEM image of transparent electrode **(f)** and high resolution top view SEM image of transparent electrode surface (Au 4 nm/PEDOT:PSS/Au 10 nm/PEM) (Scale bar = 300 nm).


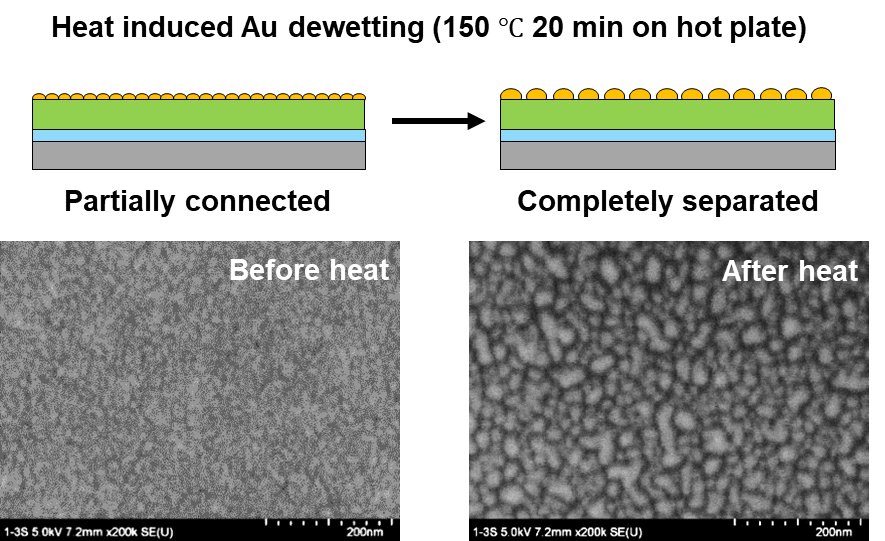


**Supplementary Figure 2.** Fabrication of independent Au island structured photothermal layer with simple heat treatment by inducing Au nanofilm dewetting. The radius of Au islands is longer after heat treatment.


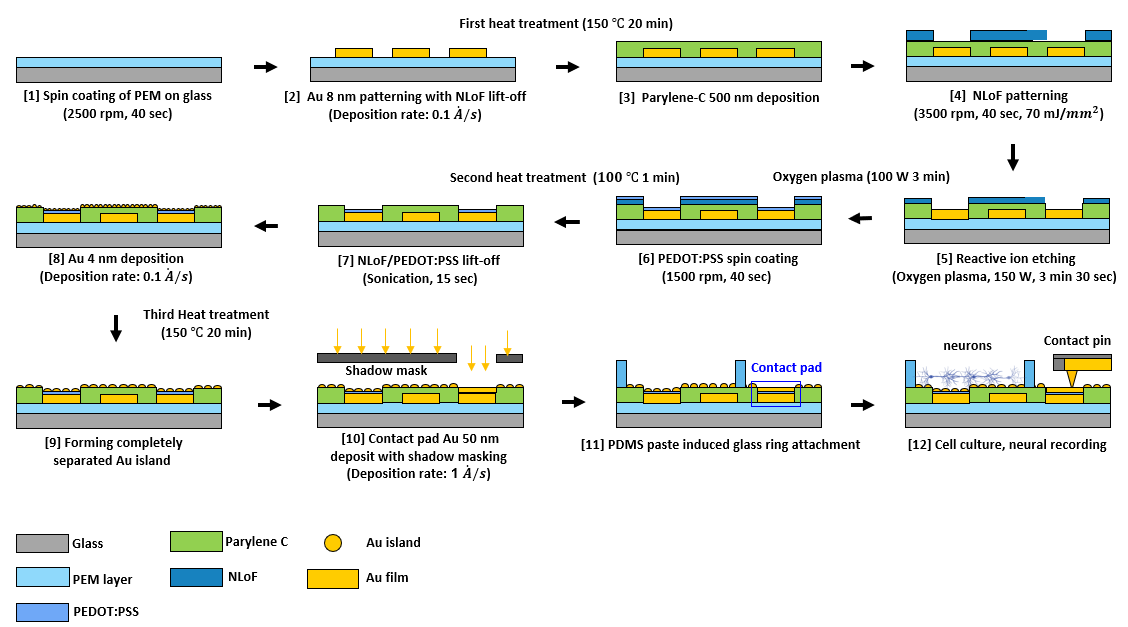


**Supplementary Figure 3. Overall fabrication steps of transparent RTD-MEA.**


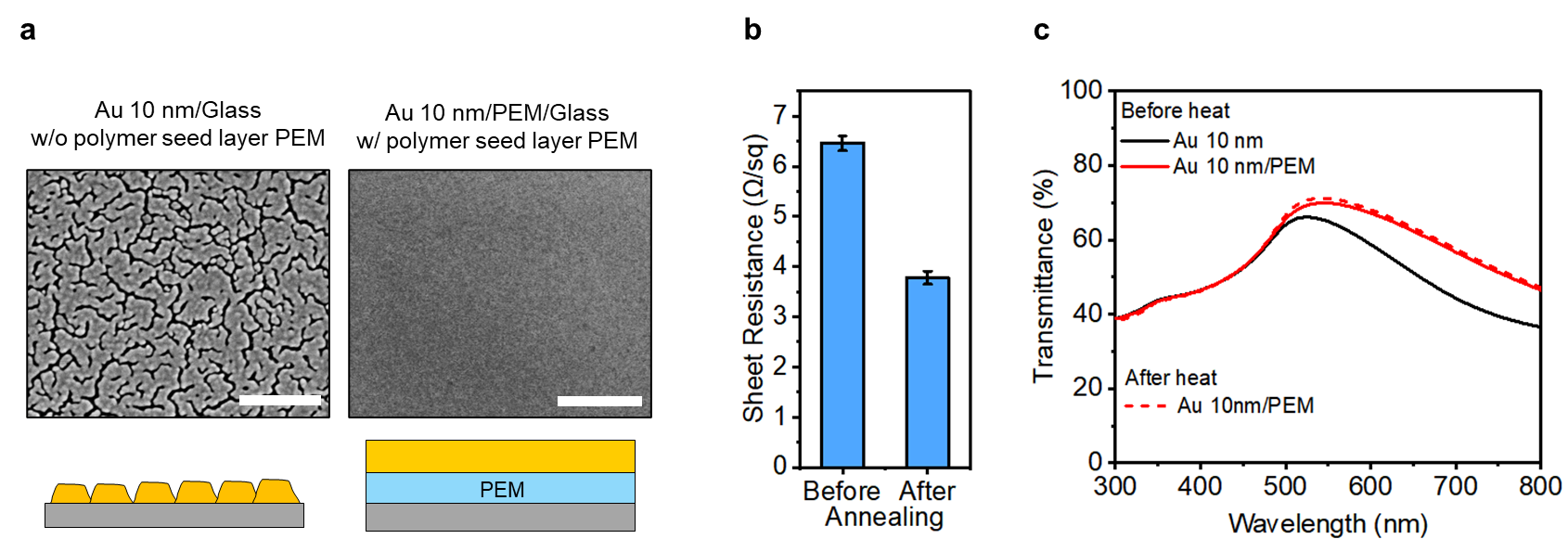


**Supplementary Figure 4.** Electrical, optical optimization of polymer seed layer induced ultrathin Au nanofilm. **(a)** SEM images of 10 nm Au films without PEM coating (left) and with pre-PEM coating (right). Both images of nanofilm are taken after 20 minutes of annealing at 150 °C on hot plate (Scale bar = 300 nm). **(b)** Sheet resistance changes before and after annealing of 10 nm Au/PEM nanofilm. **(c)** Transmittance of various samples with and without PEM seed layer and after heat treatment.


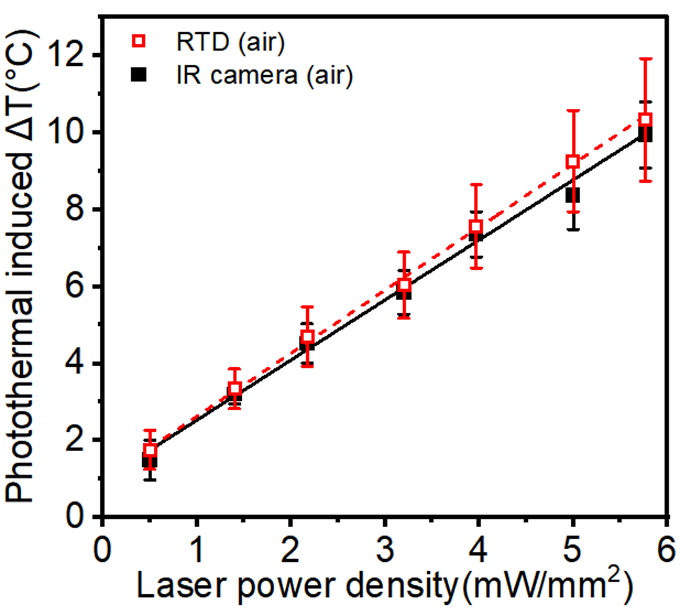


**Supplementary Figure 5. The verification of temperature synchronization between the temperature sensor (RTD) and the IR camera under air conditions.** We simultaneously measured the temperature change induced by photothermal heating using the temperature sensor in tRTD-MEA and the IR camera to assess temperature sensing performance at various NIR intensities. The average and standard deviation of the detected temperatures were calculated from three peaks observed during light-induced photothermal modulation, repeated in cycles of three on/off periods (10 s/30 s).


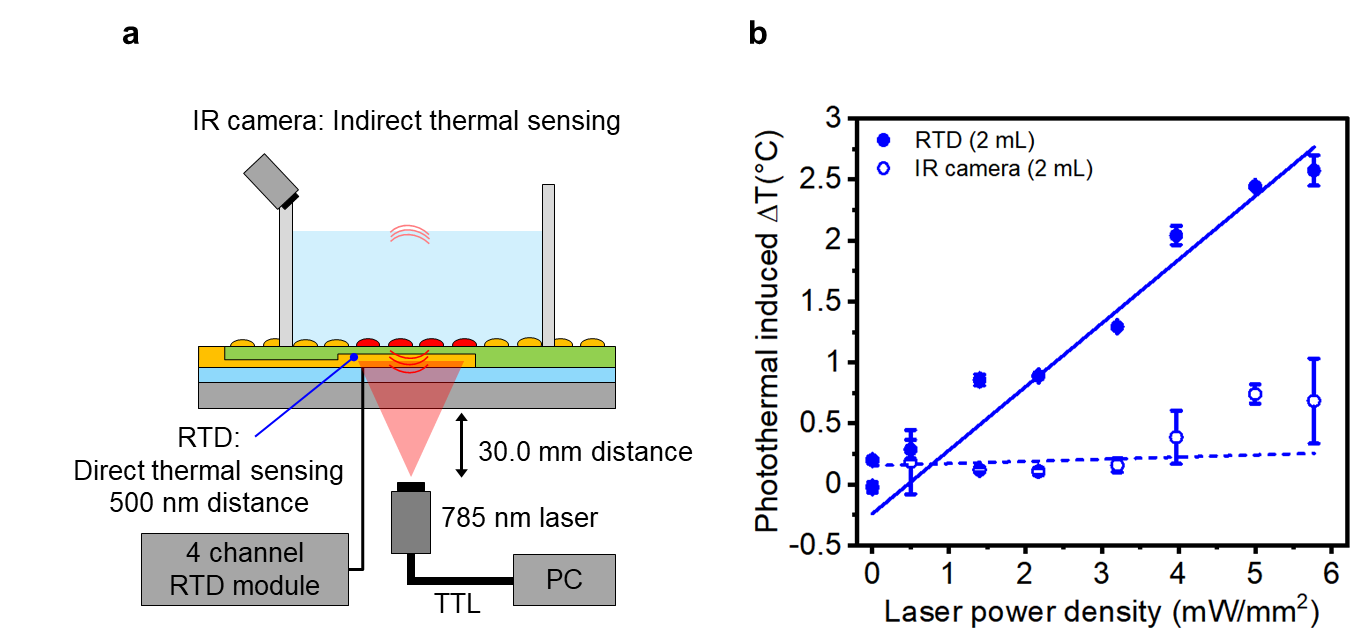


**Supplementary Figure 6. Direct sensing of photothermal-induced heat with a transparent temperature sensor in culture media. (a)** The experimental setup involves simultaneous temperature measurement using tRTD and an IR camera for photothermal-induced heat. **(b)** Photothermal-induced temperature changes at the interface in 2 mL of culture media (depths: 6.07 mm) were recorded at different NIR intensities (0-5.77 mW/mm²).


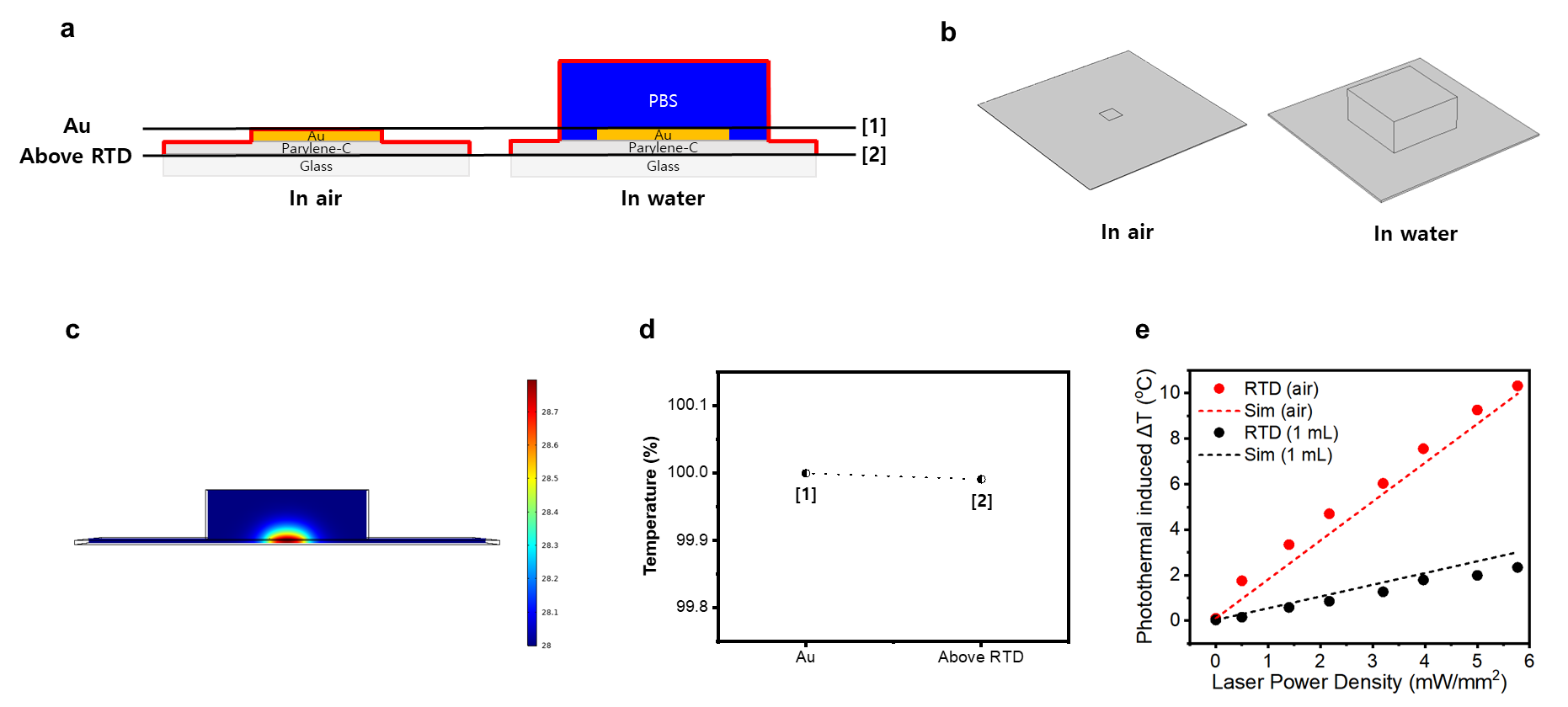


**Supplementary Figure 7. Simulation of photothermal heat-induced temperature change. (a)** Cross-section design of multimodal biosensing and tRTD-MEA in air and water. We set a thickness of 4 nm for the Au film and 500 nm for parylene-C, which are at the same scale as the real chip. The heat flux was modeled to flow along the device surface, excluding the bottom of the glass. We also marked the positions of the Au film and the transparent temperature sensor (RTD) as [1] and [2], respectively. **(b)** Modeling thermal simulation in air and water. **(c)** Computational analysis of heat distribution due to the photothermal effect in water. The heat power was set to 10 mW, and the volume of water was set to 1 mL. **(d)** Comparison of temperatures on the Au film and above the temperature sensor (RTD) as a percentage**. (e)** Comparison of the temperature changes detected by the temperature sensor (RTD) and the simulation during photothermal heat generation in air and water, respectively. The light-to-heat conversion ratio is 29%.


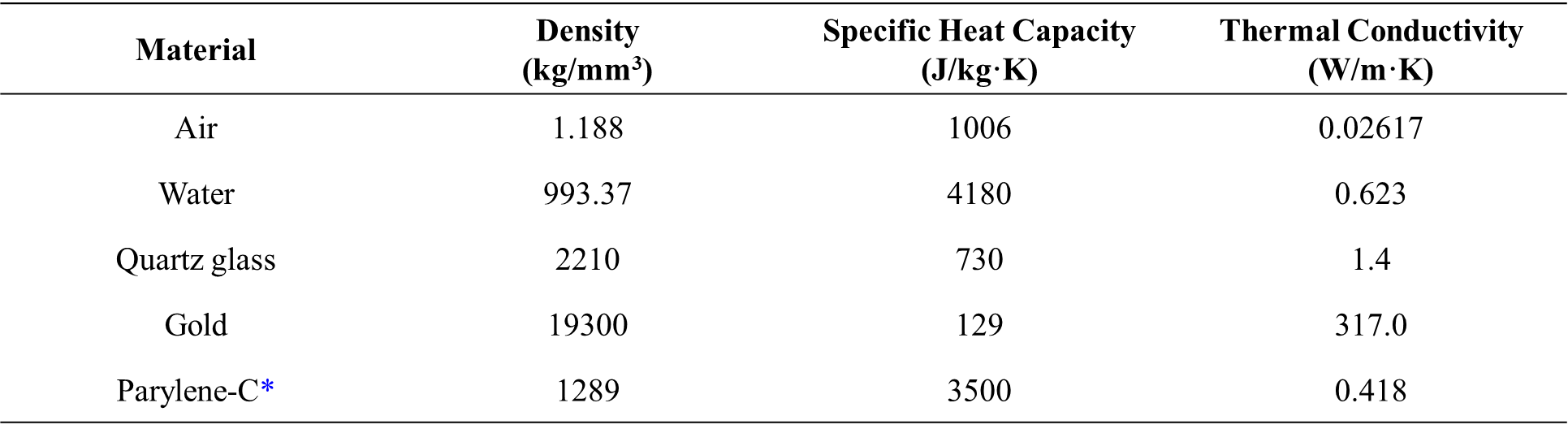


**Supplementary Table 1. Material properties for thermal stimulation used in COMSOL**

Material properties of parylene-C was adopted in a literature*, and the rest of material properties was adopted in a FEM software database.

*Guermoudi, A.A., Cresson, P.Y., Ouldabbes, A. *et al.* Thermal conductivity and interfacial effect of parylene C thin film using the 3-omega method. *J Therm Anal Calorim* **145**, 1–12 (2021).


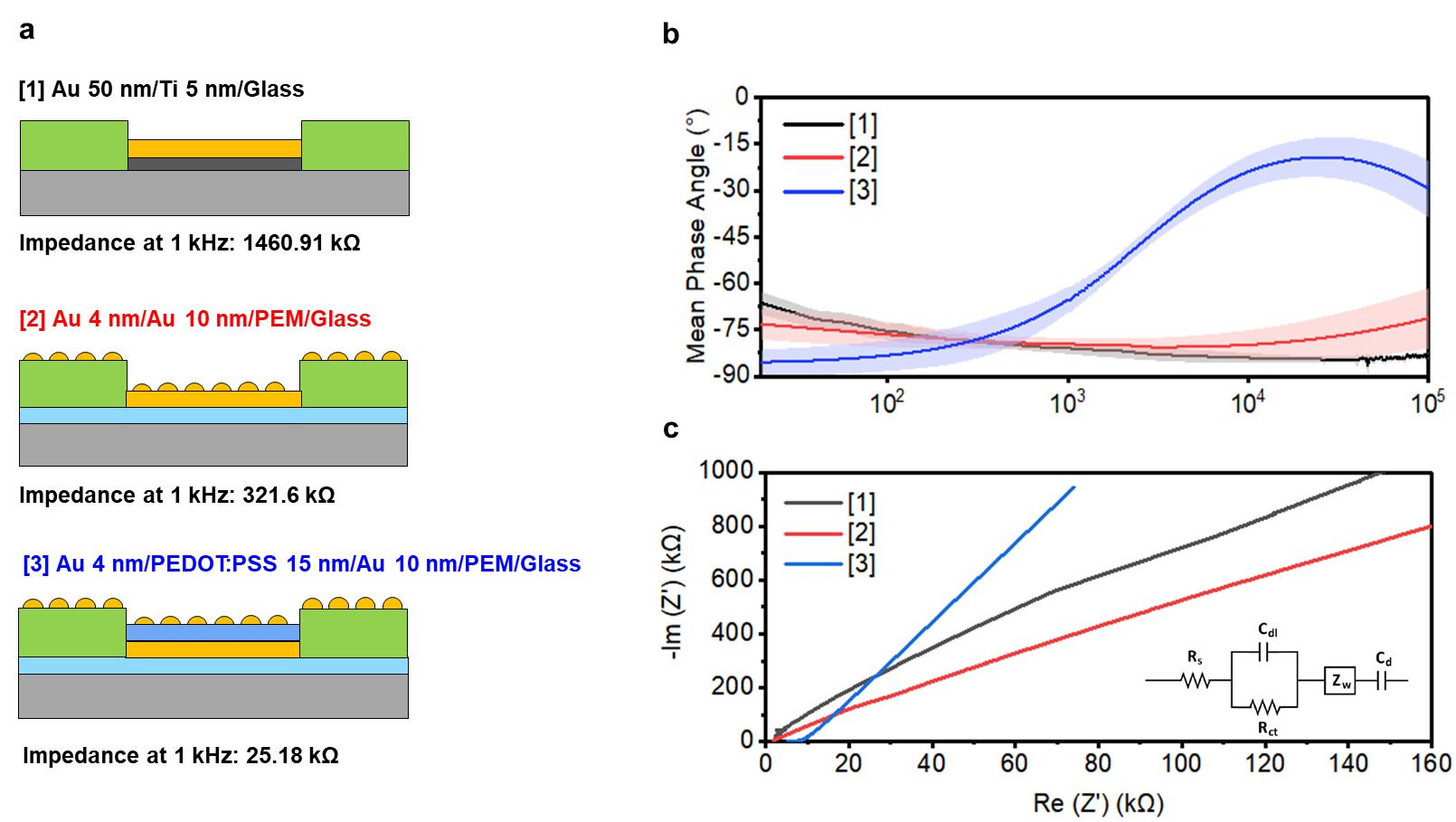


**Supplementary Figure 8. Comparison of the electrochemical properties of various types of electrodes: (a)** Cross-sectional schematic of three different types of electrodes. [1] *Au/Ti*: Au(50 nm)/Ti (5 nm), [2] *Au/PEM*: Au(4 nm)/Au(10 nm)/Polyelectrolyte multilayer (PEM), [3] *Our electrode*: Au(4 nm)/PEDOT(15 nm)/Au(10 nm)/PEM **(b)** Phase angle and **(c)** Nyquist plot with equivalent circuit of the three different types of electrodes.

We further investigated the implications of changes in the overall impedance by fitting the experimental EIS data into a simple equivalent circuit model of the microelectrode/electrolyte interface. A parallel RC circuit of a double-layer capacitor (C_dl_) and a charge transfer resistor (R_ct_) with Warburg diffusion element (Z_w_) and bulk redox capacitance (C_d_) were formed. A small resistor (Rs) was connected in series, which includes the electrolyte solution resistance and the resistance of the interconnect lines. We then fitted the experimental data of the [1] Au Ti, [2] Au 4 nm/Au 10 nm/PEM, and [3] our transparent electrode to the Randles circuit. From the Nyquist plots within the frequency range from 20 Hz to 100 kHz, our MEA shows similar Randles circuit characteristics. The results for the equivalent circuit parameters are shown in Table S2. Overall, the equivalent circuit model represented the impedance measurement data reasonably well matched with small errors of each parameter (Au/Ti: 2.00-16.47%, Au 4 nm/Au 10 nm/PEM: 2.11-14.42%, Au 4 nm/PEDOT:PSS 15 nm/Au 10 nm/PEM (Our transparent electrode): 0.17-5.45%). Noticeably, R_ct_ tended to decrease in the order of Au/Ti, Au 4 nm/Au 10 nm/PEM, our transparent electrode. Also, our transparent electrode shows the lowest resistance in the final parallel circuit. Z_w_ and 1/C_d_ also showed the same decreasing trend as R_ct_, and our transparent electrode shows the smallest values after the final sum of the three parameters. This trend can explain why our transparent electrode shows the lowest impedance.


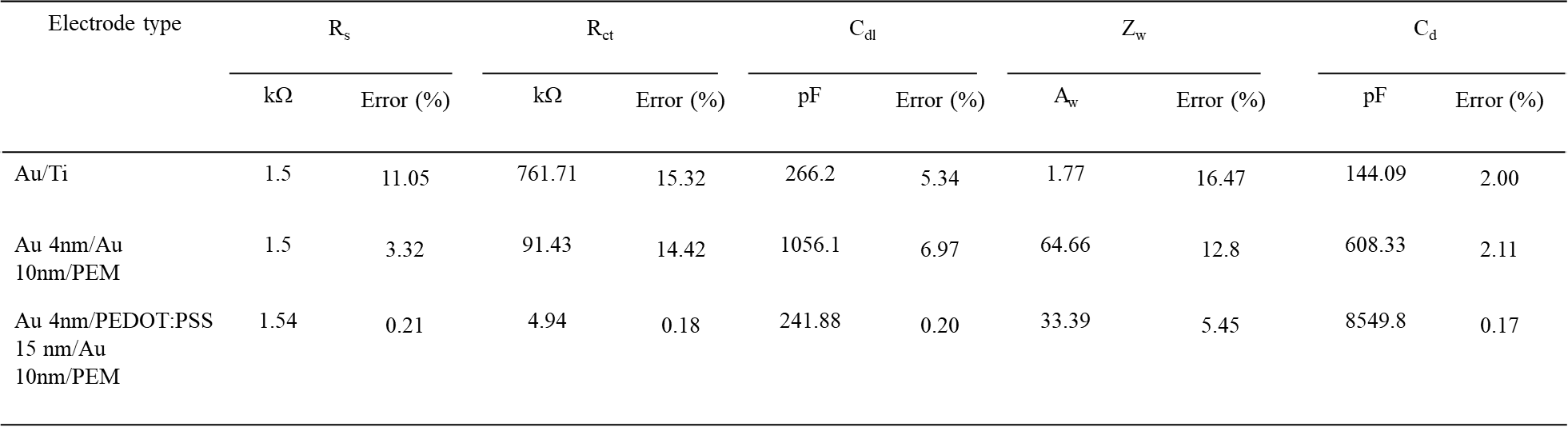


**Supplementary Table 2. Simulated parameters of the equivalent circuit model of various MEAs. (Zw = A_w_/ω^0.5^ − j A_w_/ω^0.5^).**


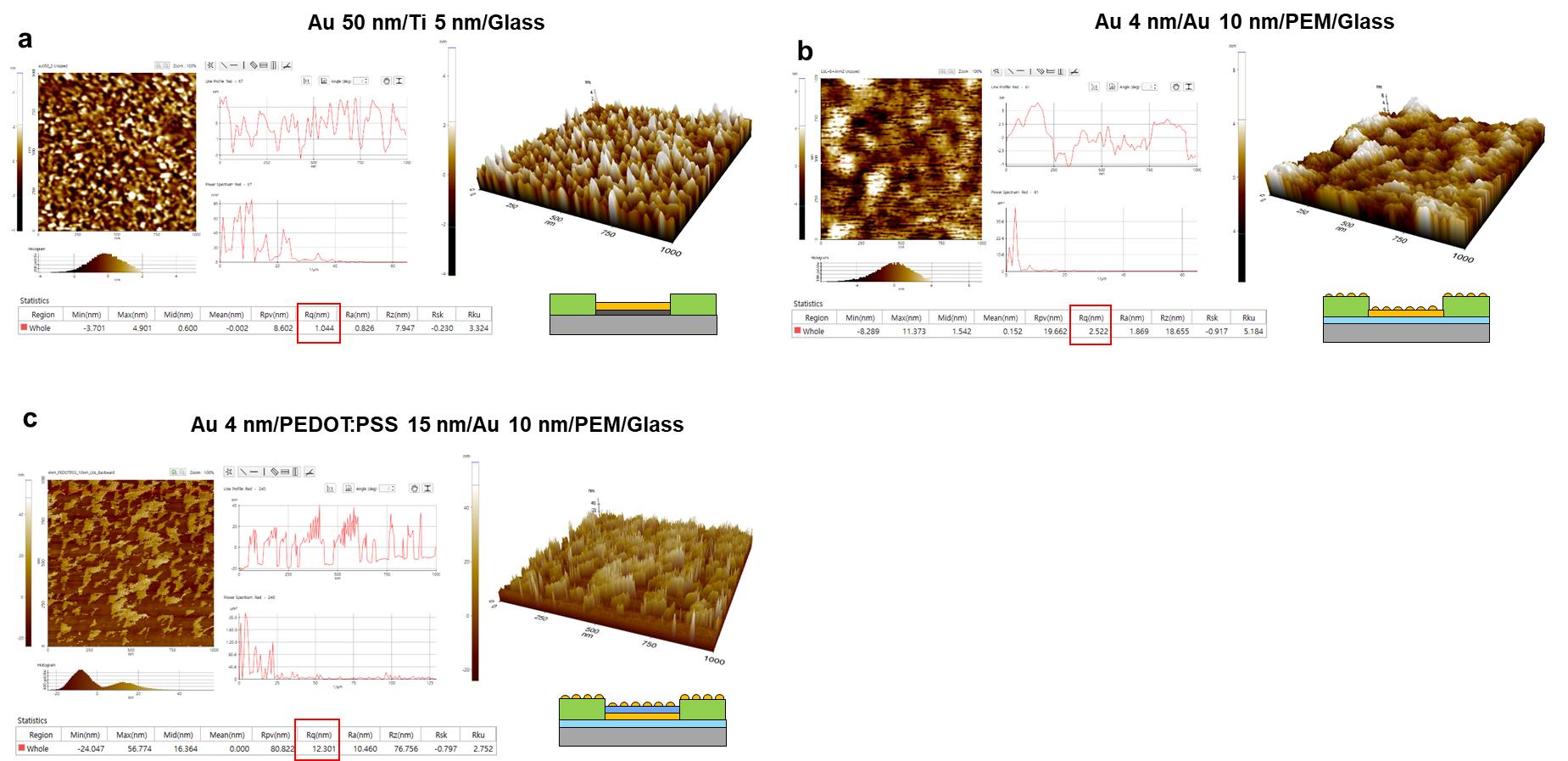


**Supplementary Figure 9. Atomic force microscopic (AFM) images of various types of electrode surfaces.** 2D and 3D images with quantitative analysis data (scanning area: 1 μm × 1 μm, 256-bit × 256-bit): **(a)** Au/Ti, **(b)** Au/PEM, and **(c)** Our electrode: Au 4 nm/PEDOT:PSS 15 nm/Au10 nm/PEM.


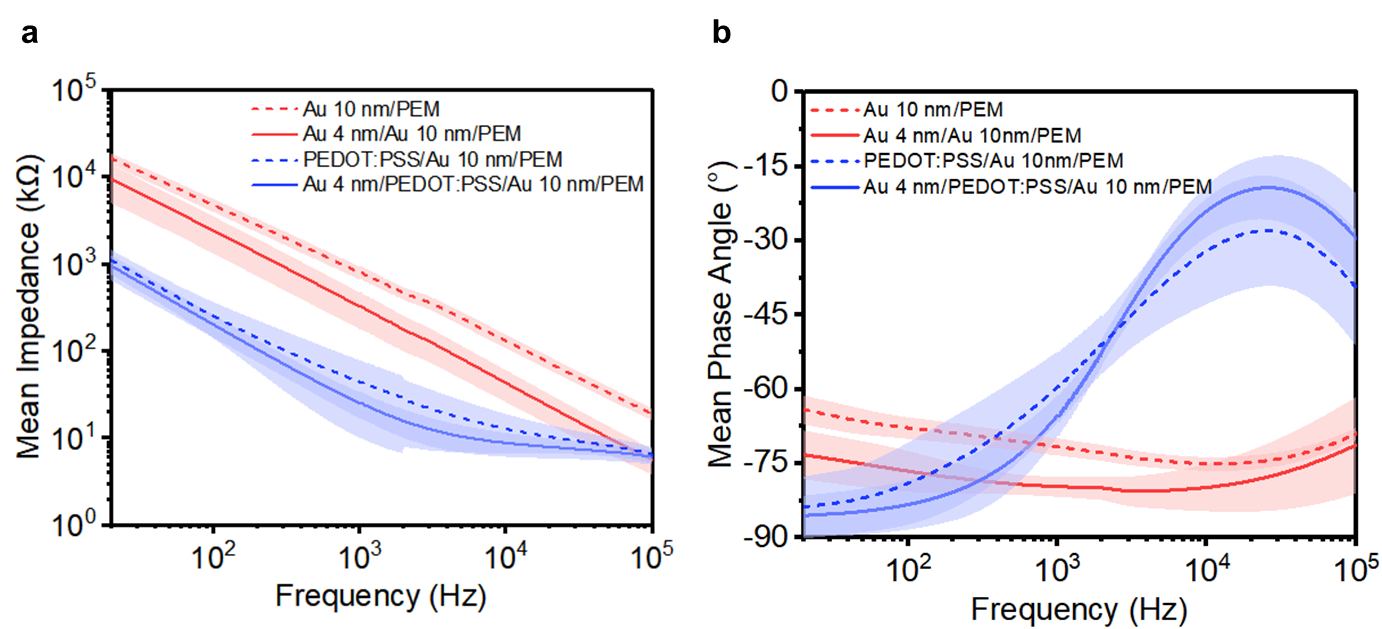


**Supplementary Figure 10. The effects of a top layer of 4 nm Au islands on the electrochemical properties of electrodes. (a, b)** Impedance and phase angle changes of each electrode depend on the presence of the top layer of 4 nm Au islands (Before: dashed lines, After: solid lines). The total number of measured electrodes is indicated (blue: 16, red: 21).


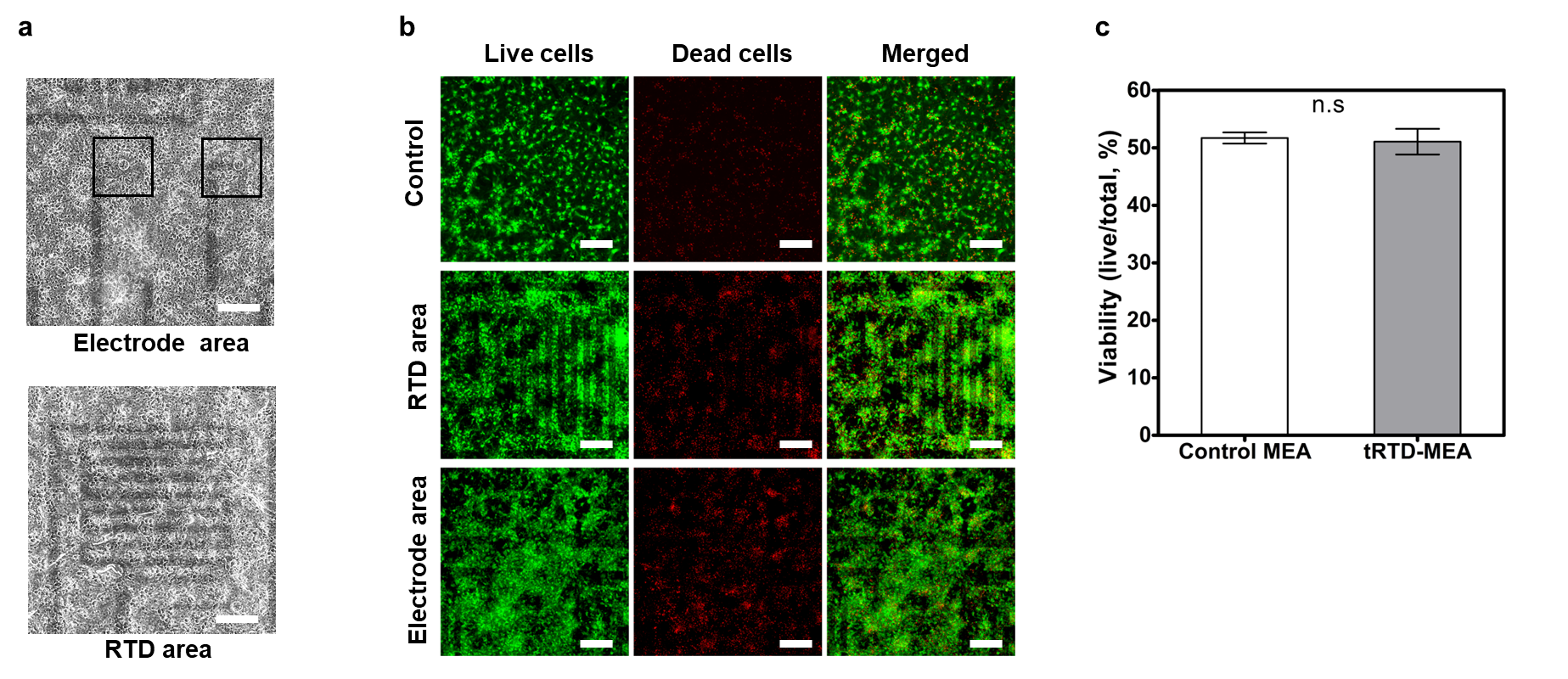


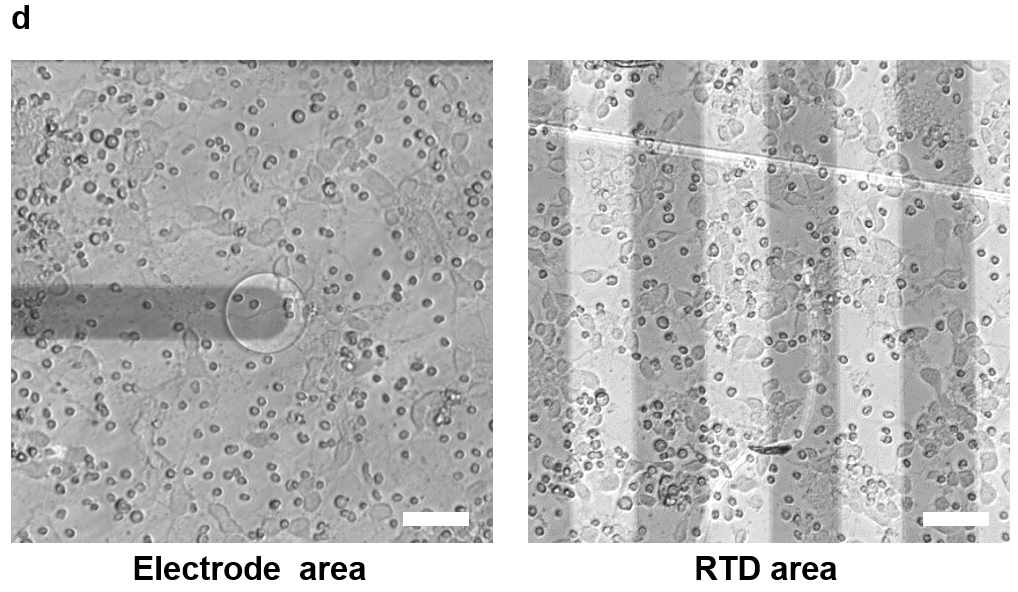


**Supplementary Figure 11. Phase contrast imaging and cell viability test. (a)** Phase contrast images of hippocampal neurons on the electrode area and RTD area of the tRTD-MEA (Scale bar = 200 µm). **(b)** Live/DEAD assay was performed the biocompatibility test between 14-21 DIV cultured neurons on the tRTD-MEAs and control MEAs. Live cells are indicated by the green fluorescence, while dead cells are indicated by the red fluorescence. **(c)** All procedures counted dead cells by applying neuron nucleus size using ImageJ. The viability of neurons cultured with control MEAs and tRTD-MEAs was analyzed by unpaired t-test (n = 4 ROIs from 3 chips each, scale bar = 200 µm). The dead cells in both MEAs were mostly those that died during the seeding process in the primary culture, while the growing cells attached to the tRTD-MEAs grew as well as the control MEAs. The viability results were tested statistically using an unpaired one-tailed t-test. There was no significant difference between samples and the P value was 0.4145. **(d)** The transparency of the electrode area and RTD area allowed for imaging of the neural cells on the device. (Scale bar = 50 µm).


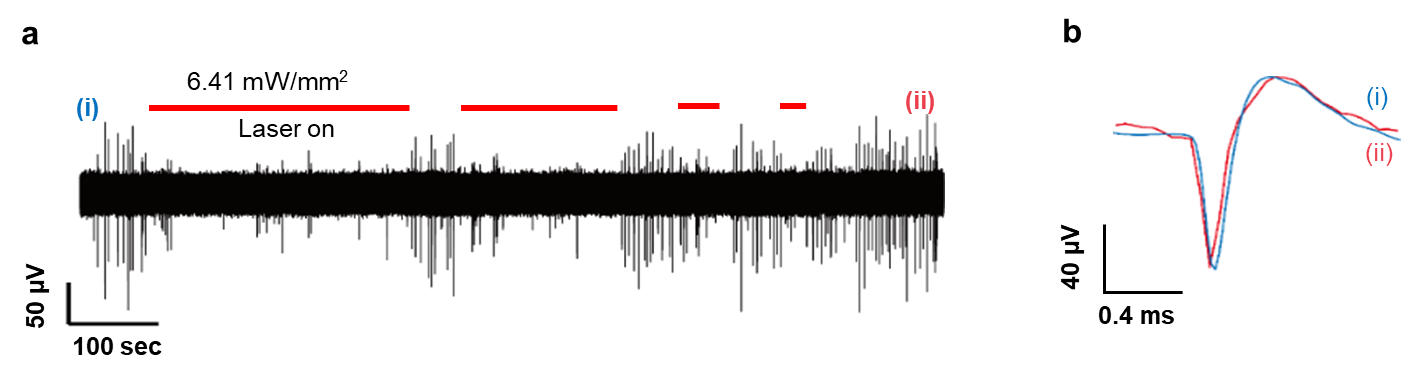


**Supplementary Figure 12. Neural spike inhibition during NIR irradiation on tRTD-MEA. (a)** A neural spike activity trace for different NIR irradiation durations (Laser power density: 6.41 mW/mm^2^). **(b)** Comparison spike waveform before and after the photothermal stimulation.
